# Characterisation of Studies on Consumers’ Home Food Safety Knowledge, Attitudes, and Practices (KAP): A Scoping Review

**DOI:** 10.3390/foods15101730

**Published:** 2026-05-14

**Authors:** Antonella Maugliani, Monica Valli, Francesca Maialetti, Francesca Baldi, Cinzia Civitareale, Manuela Luzi, Manlio Mammoli, Duilio Luca Bacocco, Donatella Gentili, Francesca De Battistis

**Affiliations:** 1 Department of Food Safety, Nutrition and Veterinary Public Health, Istituto Superiore di Sanità, Viale Regina Elena 299, 00161 Rome, Italy; francesca.maialetti@iss.it (F.M.); francesca.baldi@iss.it (F.B.); cinzia.civitareale@iss.it (C.C.); francesca.debattistis@iss.it (F.D.B.); 2 National Centre for Disease Prevention and Health Promotion, Istituto Superiore di Sanità, Viale Regina Elena 299, 00161 Rome, Italy; monica.valli@iss.it; 3 IT Unit, Organisation of the Central Directorate for General Affairs, Istituto Superiore di Sanità, Viale Regina Elena 299, 00161 Rome, Italy; manuela.luzi@iss.it (M.L.); manlio.mammoli@iss.it (M.M.); duilioluca.bacocco@iss.it (D.L.B.); 4 Scientific Communication Service, Library, Istituto Superiore di Sanità, Viale Regina Elena 299, 00161 Rome, Italy; donatella.gentili@iss.it

**Keywords:** consumer behaviour, home food safety, knowledge attitudes practices (KAP), scoping review, surveys, high-income countries

## Abstract

Home food safety (HFS) is a major contributor to foodborne illness, often originating in domestic settings. Although population-based studies using surveys, questionnaires, and interviews are commonly used to assess consumers’ HFS-related knowledge, attitudes, and practices (KAP), methodological heterogeneity limits comparability across studies. This scoping review aimed to map studies assessing consumers’ HFS-related KAP in high-income countries, describe recurrent methodological and reporting features, and identify areas of variability. Following the Arksey and O’Malley framework and JBI guidance, the literature published between 2000 and 2023 was systematically searched across five scientific databases, as well as governmental and institutional sources for the grey literature. Data extraction and synthesis were guided by an expanded 15-feature framework refined from a previous rapid review. A total of 274 documents were included (247 scientific articles and 27 governmental and institutional reports). Across the included studies, several methodological features showed high consistency, including primary data collection (93%), predominantly cross-sectional designs (91%), the use of closed-ended instruments (71%), quantitative analytical approaches (78%), and voluntary, non-incentivised participation (68%), suggesting the presence of a common descriptive methodological core. At the same time, substantial variability was observed in sample size (62%), study aims (52%), analytical strategies (52%), modes of administration (51%), geographic coverage (47%), thematic scope (44%), and study period (54%). The coexistence of methodological convergence and context-dependent variability poses challenges in terms of evidence synthesis and comparability in HFS-related KAP research. The 15-feature framework developed in this review provides a structured, non-prescriptive tool to support transparent description and comparison of methodological and reporting practices. By pinpointing common approaches and areas of divergence, this review offers a foundation for guiding future HFS-related KAP research and supporting the development of more comparable and policy-relevant evidence.

## 1. Background

### 1.1. Food Safety, Burden of Foodborne Illnesses

Food safety (FS) refers to practices relating to the handling, storage, and preparation of food that aim to prevent exposure to food-related hazards and protect consumers from foodborne illness (FBI). These hazards include microbiological agents, chemical contaminants, and other factors capable of producing adverse health effects following food consumption.

The Codex Alimentarius Commission defines risk as “a function of the probability of an adverse health effect and the severity of that effect, consequential to a hazard(s) in food” [1].

In FS settings, risks are traditionally classified as microbiological or chemical. Microbiological risks concern the presence of microorganisms and their toxins with adverse effects on human health. Chemical risks are also an important source of FBI, though its attribution to a specific food can be challenging [2].

From a broader public health perspective, some studies also address nutritional risks, defined as the probability of adverse health effects associated with inadequate energy or nutrient intake over an extended period, as a result of household-level dietary habits and practices. In this review, nutritional aspects are discussed in relation to domestic food-related practices, in addition to conventional microbiological and chemical food safety risks.

A substantial proportion of FBI can be attributed to improper food management in domestic environments. More than two decades ago, Redmond and Griffith [3] emphasised the role of consumer behaviour at home as a critical determinant of foodborne disease risk. Subsequent studies have estimated that more than 30% of all cases of foodborne disease may be associated with the domestic setting [4]. Symptoms such as diarrhoea are often perceived as transient and seldom attributed to inadequate hygiene practices at home [3,5].

The World Health Organization has consistently emphasised the importance of the household as a crucial point of exposure along the food chain, particularly given the loss of control that occurs after purchase and during the domestic handling, storage, and preparation stages [2]. Although precise root cause attribution varies depending on regional surveillance practices and data sources, evidence from high-income settings is consistent with this view, as is demonstrated, for example, by the finding that 29.8% of reported foodborne outbreaks occurring in the European Union in 2022 were associated with private households [6].

### 1.2. Importance of Home Food Safety Behaviours and Perceptions

FS can be compromised at various stages along the food chain, and while the food industry business is under an obligation to follow strict, regulation-compliant processes to minimise risks within the ‘farm to fork’ framework, private individuals are not required to observe similar procedures from retail to consumption [7]. Since domestic food handling consists primarily of individual behaviour and informal routines, consumers are responsible for risks within the domestic setting; however, even when they possess FS knowledge, they do not necessarily apply safe food-handling practices at home. Risk underestimation and inconsistent application of safe behaviour are key barriers to improving HFS. The availability of clear, reliable information regarding the likelihood and consequences of consumption-related hazards enables better-informed choices and safer behaviour [8,9,10], making effective disclosure by public health organisations, scientific authorities and governments—at both national and regional levels—essential.

In this review, HFS is operationally defined as food-related behaviour occurring in domestic settings, including handling, storage, preparation, and practices associated with hygiene (e.g., handwashing, cleaning and dishwashing), prevention of cross-contamination, temperature control, refrigerator organisation, and management of leftovers, as well as other daily food-related practices discussed in the included studies.

### 1.3. Importance of Recommendations, Food Health Authorities

Several consumer-oriented FS recommendations and guidelines have been developed to promote safe food handling, storage, and preparation practices in the home environment [11,12,13,14,15]. Although it is not possible to achieve industrial-level control in home environments, clear and accessible guidance is crucial, since consumers often overlook safe practices because they underestimate the associated risks. The need for effective educational initiatives to promote safer attitudes and behaviour has been advocated for decades and remains an active area of research [8,16,17,18,19].

Assessing consumers’ KAP in domestic settings is therefore valuable for guiding targeted health-promotion strategies. More than two decades after foundational reviews such as the review by Redmond and Griffith [3], research continues to investigate the determinants of unsafe food-handling behaviours in the home, highlighting the persistent need to strengthen the production of actionable evidence and develop and refine effective risk-communication strategies [10,17,20,21].

### 1.4. Assessing Consumers’ Knowledge, Attitudes, and Practices in Home Settings

Population-based studies employing surveys, questionnaires, interviews, and related data collection approaches are an essential means of assessing baseline consumer knowledge and routine domestic food-handling practices. Such studies also support the identification of knowledge gaps and the development of targeted risk-communication initiatives to promote health [4,19,22,23]. In order to generate reliable evidence, these studies need to provide adequate methodological detail, ensure reproducibility, and emphasise sources of bias [24]. Previous studies have emphasised the need for basic instrument development criteria [25] and shown that heterogeneous measurement scales and data-collection methods hinder comparability across studies [26,27,28]. From a temporal perspective, Bird (2009) [25] emphasised the importance of using clear questionnaire templates for questionnaires assessing public knowledge and perceptions. Fein et al. (2011) [26] subsequently observed that, despite more than two decades of published studies on food-handling behaviour and high-risk consumption practices, the use of non-comparable study designs had resulted in relatively few trend analyses being carried out. More recently, Shelley et al. (2021) [24] reported that although guidance on good questionnaire practice exists, it is often inadequately applied. In 2023, we contributed to this research area by characterising survey structures used in the FS literature by means of a rapid review focusing on pregnant women [29].

In our review, we found comparability across studies assessing consumers’ KAP to be hindered by study design diversity, considerable methodological heterogeneity, and variability between countries. To improve the characterisation of this evidence base, we conducted a scoping review, the most appropriate approach for mapping the breadth, depth, and nature of existing research. This review aimed to summarise scientific publications and governmental and institutional reports employing population-based studies to assess HFS-related KAP in the general population of high-income countries and to identify methodological gaps. Compared to the preceding rapid review, this study expanded the search to five scientific databases, incorporated the grey literature (governmental and institutional documents), and extended the timeframe to include studies published between 2000 and 2023. A preliminary search identified no similar registered scoping reviews, which supports the relevance of this project.

Hence, this scoping review aimed to identify the key methodological and reporting features of studies assessing consumers’ HFS-related KAP in high-income countries, with a view to promoting greater homogeneity and comparability and strengthening the evidence base for effective public communication and FS literacy strategies. High-income countries were selected because the FS challenges in low- and middle-income settings are considerably different [29,30], thereby limiting cross-context comparability, since clear interpretation is more likely in relatively similar socio-economic and regulatory environments.

## 2. Methodology

We conducted a scoping review of peer-reviewed and grey literature documents adressing consumers’ food safety-related knowledge, attitudes, and practices (KAP) in home settings. The review included documents published in high-income countries between January 2000 and August 2023. The final literature search was completed on 3 August 2023.

To define a high-income country, we adopted the World Bank classification based on Gross National Income (GNI) per capita data (World Bank Classification, https://datatopics.worldbank.org/world-development-indicators/the-world-by-income-and-region.html accessed on 27 June 2024).

The scoping review followed the methodological framework originally proposed by Arksey and O’Malley [31] and subsequently enhanced by Levac et al. [32] and Peters et al. [33]. Reporting complied with the PRISMA-ScR guideline [34], including the PRISMA-ScR checklist of 20 essential and two optional items [35] (see Supplementary Materials S1). The review comprised five stages: (1) research question definition; (2) relevant studies searches; (3) study selection; (4) data charting; and (5) results collating, summarising and reporting.

The study protocol was prospectively registered on the Open Science Framework on 1 June 2022 (OSF registration link: https://osf.io/p6xhw accessed on 16 March 2026).

The interdisciplinary review team collaboratively developed the protocol. The research questions, search strategy and eligibility criteria were guided by the Population–Concept–Context (PCC) framework (Table 1).

### 2.1. Research Question(s) Definition

Main Research Question: What are the key methodological and reporting features of studies assessing consumers’ KAP on HFS?

Sub-questions: (a) What are the main methodological differences and similarities across studies? (b) Is there evidence of a standardised approach or protocols for surveys assessing consumers’ KAP on HFS? (c) Do studies address a specific target population (e.g., age, vulnerability) or geographic context?

### 2.2. Relevant Studies—Research Strategy

Below, we report the methodologies by the type of literature, divided into subsections.

#### 2.2.1. The Peer-Reviewed Literature

Search strategies were developed in collaboration with an expert librarian (D.G.) using controlled vocabulary terms (e.g., Medical Subject Headings -MeSH, Emtree) and free text keywords in line with the PCC framework. Searches were conducted in five electronic databases: MEDLINE (via PubMed), Embase, Scopus, Web of Science, and the Cochrane Library.

The search was completed on 3 August 2023 and was restricted to articles published between January 2000 and August 2023. Full search strategies for all databases are provided in Supplementary Materials S2.

#### 2.2.2. The Grey Literature

The grey literature was identified using two separate and complementary approaches:

- Governmental documents: these documents were identified using Google Advanced Search (https://www.google.com/advanced_search accessed on 10 May 2026), which was accessed through the Google Chrome web browser in incognito mode. To mitigate potential biases related to search personalisation and temporal variability, all searches were conducted without an active Google account, clearing caches and cookies before each search session, and using predefined and consistent search strings. Search strategies were in line with the PCC framework and refined with the support of IT specialists (M.L., D.L.B., M.M.).
  For each high-income country, up to three food safety-related governmental websites were searched. For European Union Member States, the websites were selected based on the EU Food Safety Almanac [36]. For non-EU high-income countries, searches targeted the Ministry of Health, the national food safety authority, and the national public health institute (or equivalent body). Searches were primarily conducted in English to ensure consistency across countries. Additional searches in Italian, Spanish, and Chilean Spanish were run to leverage the language expertise of the review team and enhance the retrieval of governmental documents that are not systematically published in English in those specific national contexts. All governmental searches were completed on 17 October 2023. Full search strings and the complete list of websites searched are reported in Supplementary Materials S3.
- EFSA Journal: Given its role as the official repository of the European Food Safety Authority’s scientific outputs on food and feed risk assessment—including nutrition, animal and plant health, and plant protection products—the EFSA Journal was searched separately (https://efsa.onlinelibrary.wiley.com/journal/18314732 accessed on 10 May 2026). Keyword combinations and Boolean operators reflecting the same PCC concepts used for the other search strategies were applied. The EFSA Journal search was conducted in September 2023. Although the EFSA Journal is a peer-reviewed scientific journal, it was treated as an institutional source within the grey literature workflow of this review, in order to systematically capture EFSA’s agency-level outputs. This methodological choice is being explicitly reported for transparency and scope alignment purposes.

### 2.3. Eligibility Criteria

Eligibility rules were applied to refine selection for both the scientific and grey literature. Studies were included if all inclusion criteria were met and excluded if any exclusion criteria applied (Table 2).

### 2.4. Study Selection

#### 2.4.1. The Peer-Reviewed Literature

The search results were exported to EndNote Web (v. 21; EndNote Web/Online/Basic Comparison), for initial duplicate removal and harmonisation of RIS file formats before import into Covidence (Systematic review software, Veritas Health Innovation, Melbourne, Australia. Available at www.covidence.org). Covidence supported collaborative screening and decision documentation and ran an additional automated de-duplication. Study selection consisted of a two-stage process based on predefined eligibility criteria: (i) title and abstract screening and (ii) full-text assessment. Records were allocated to two independent reviewer pairs (Team 1: F.B., C.C.; Team 2: F.M., F.D.B.). Discrepancies were resolved through discussion with a third reviewer (A.M.) and, when required, with the broader review team to reach consensus. Formal inter-rater reliability statistics (e.g., Cohen’s kappa) were not calculated, as the scoping review followed a consensus-based approach; however, independent screening and structured consensus resolution were applied to ensure consistency and reliability in study selection.

#### 2.4.2. The Grey Literature

The same two-stage screening approach (title and abstract screening followed by full-text assessment) was applied to grey literature documents. Records were managed in Microsoft Excel to track screening decisions and mirror the transparency of the Covidence workflow. Two reviewers (F.M., M.V.) independently screened all documents. Disagreements were resolved by consensus with a third reviewer (A.M.) and, when necessary, with the full review team. IT specialists supported data management and ensured data integrity throughout the process.

### 2.5. Data Charting—Data Extraction

A data extraction template was collaboratively developed by the review team. The template was initially created within Covidence and independently piloted by both reviewer teams on a purposive sample of 10–15 peer-reviewed studies. The piloting phase was conducted using the eight key features adopted in the authors’ previous rapid review [29], in order to assess their applicability to a broader and more heterogeneous body of evidence.

The piloting exercise showed that the initial set of eight key features did not capture the full range of methodological and reporting characteristics observed across the included studies. To ensure a more granular and consistent characterisation prior to full data extraction, the set of key features was therefore expanded.

Feedback from the piloting phase was discussed collectively, and the data extraction form was refined through an iterative process until consensus was reached on the structure and variables to be charted. This refinement process was completed prior to the extraction of the full set of included studies. Once finalised, the data extraction form was exported to Microsoft Excel and applied to all included sources, encompassing both the scientific and grey literature, for subsequent analysis. Each row represented an individual paper or report, while the columns represented the predefined variables extracted for the review (see Supplementary Material S4).

### 2.6. Data Analysis—Result Collating, Summarising and Reporting

Data analysis focused on a set of key features deemed relevant to addressing the review questions and systematically describing the methodological and reporting features of the included studies. These features initially consisted of the eight key features identified in a previous rapid review conducted by some of the authors [29] and were subsequently refined, as described above, prior to full data extraction.

Most extracted data were synthesised narratively; where appropriate, simple descriptive statistics (e.g., frequencies and percentages) were used to summarise patterns across the included studies and to support the presentation of the results. No inferential statistical analyses were performed. In keeping with the objectives of this scoping review, no formal critical appraisal of methodological quality was undertaken, as the purpose of the review was to characterise study methodologies and reporting practices, rather than to evaluate effect estimates.

## 3. Results

**Figure 1:**
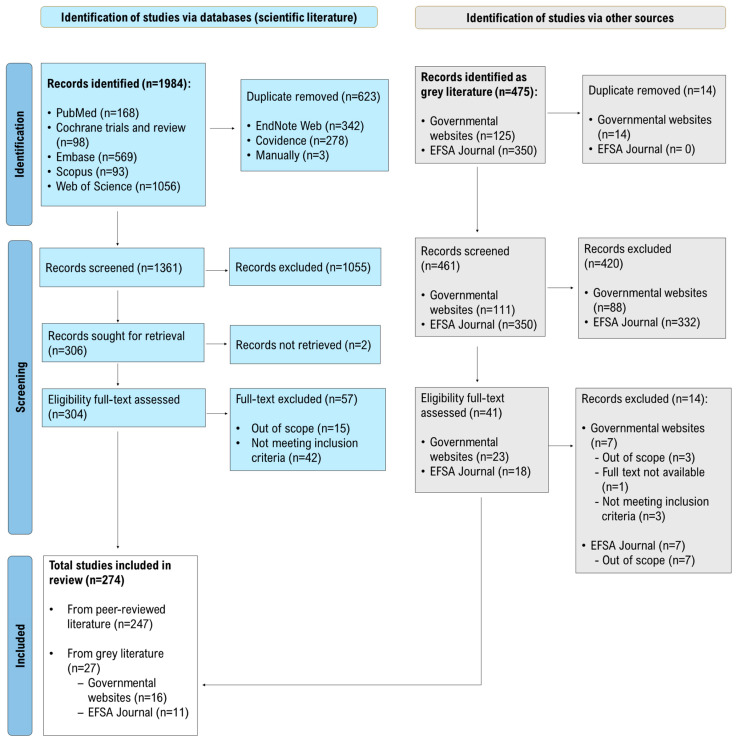
PRISMA-ScR flow diagram (adapted from [37]). Flow diagram illustrating the identification, screening, eligibility assessment, and inclusion of studies from the scientific and grey literature sources. Colour coding distinguishes the scientific literature (blue) from the grey literature (grey), which includes governmental documents and EFSA Journal reports identified and screened through separate selection flows. The final inclusion box (white) reports the total number of studies included in the review, with their distribution by source. Numbers represent records and documents at each stage of the selection process. Reasons for exclusion were grouped into broader categories for clarity. Percentages reported in the Results section for the grey literature refer to the respective subgroups (i.e., governmental documents and EFSA Journal records) and were calculated using the subgroup.

The results of the search, screening, and study selection processes are presented below. The study selection process is summarised in the PRISMA ScR flow diagram (Figure 1), which illustrates the identification, screening, eligibility assessment, and inclusion of the documents, making a distinction between the scientific and grey literature.

### 3.1. Study Selection

The scientific literature search identified 1984 records across five electronic databases. Following the removal of 623 duplicates, 1361 records remained for title and abstract screening. At the identification stage, the contribution of each database before and after duplicate removal was as follows: PubMed (168 records initially identified; 102 retained after deduplication), Cochrane (98 initially identified; 95 retained), Embase (569 initially identified; 368 retained), Scopus (93 initially identified; 73 retained), and Web of Science (1056 initially identified; 723 retained).

Of the 1361 records screened at the title and abstract level, 1055 records (77.5%) were excluded as clearly irrelevant to the review question based on the predefined eligibility criteria. A total of 306 full-text articles were assessed for eligibility (2 articles were not retrieved), leading to the exclusion of 57 records (18.7%). The main reasons for exclusion at the full-text stage, grouped into broader categories for clarity, are summarised in the PRISMA ScR flow diagram (Figure 1). A total of 247 scientific studies were included.

The grey literature searches yielded 475 records, originating from governmental sources and the EFSA Journal. Among the 125 governmental documents, 14 duplicates were manually removed. Following title and abstract screening, 88 documents (79.3% of governmental records) were excluded as irrelevant. The full-text assessment of the remaining 23 documents resulted in the exclusion of 7 records (30.4%), and therefore, 16 governmental documents were included.

The EFSA Journal search yielded 350 records. After title and abstract screening, 332 documents (94.9% of the EFSA Journal records) were excluded. Of the 18 records assessed at the full-text level, 7 (38.9%) were excluded, and therefore, 11 EFSA Journal reports were included.

A total of 274 documents were included for data extraction, 247 scientific studies and 27 grey literature documents (16 governmental documents and 11 EFSA Journal reports). The full list of included documents is provided in Supplementary Material S5.

Based on the included documents, 15 key features were identified and analysed, as described below.

### 3.2. Key Features

The analysis of the 274 included documents identified 15 key features, which are summarised in Table 3.

For each feature, the table shows the total number of documents in which the feature was identified, stratified by the scientific and grey literature, together with the corresponding operational categories.

- Key feature 1—Article type. Studies were classified as primary data collection (labelled as experimental), secondary analysis (labelled as observational), or other. Approximately 93% of the documents involved primary data collection. A total of six review and meta-analysis articles were included in the present scoping review.
- Key feature 2—Data collection method. Data collection methods were categorised as cross-sectional, longitudinal, retrospective, or other. Most of the studies employed a cross-sectional design (91%).
- Key feature 3—Testing. The use of pre- and/or post-intervention assessments to evaluate HFS-related KAP was classified as Yes, No, or Not applicable. Overall, testing was not conducted in approximately 84% of the documents, showing that cross-sectional study designs were predominant in the reviewed literature.
- Key feature 4—Data analysis method: Data analysis approaches were classified as qualitative, quantitative, semi-quantitative, or mixed. Approximately 78% of the documents reported quantitative analyses.
- Key feature 5—Target population. Studies were grouped into three mutually exclusive population categories: adults, vulnerable populations, and representative sample groups. The adult category included studies targeting the general adult population when no specific population subgroup was explicitly defined by the authors. This category could therefore encompass heterogeneous adult participants, including elderly individuals or volunteers with various characteristics, when these were not identified as the specific focus of the study. The vulnerable populations category included studies explicitly targeting defined vulnerable groups, such as elderly individuals, immunocompromised persons, patients with chronic diseases, and pregnant or postpartum women. Representative sample groups comprised studies focusing on specific population segments, including students, adolescents, and defined ethnic or community groups (e.g., American Indian and Alaska Native Navajo communities or Australian Aboriginal populations). Overall, approximately 73% of the documents focused on adults, 20% on vulnerable populations, and 7% on representative sample groups.
- Key feature 6—Question types. Question formats were classified as closed, open, semi-closed, mixed, not reported, or other. Closed-ended items included both binary or multiple-choice questions and Likert-type items for the purpose of descriptive classification. Overall, closed-ended items were reported in approximately 71% of the documents.
- Key feature 7—Cash or voucher compensation. Participant compensation was classified as reported, not reported, or not available when this information was not provided in the study. In 68% of studies, no compensation was reported.
- Key feature 8—Sample size. Studies were grouped into five categories: ≤1000; 1000–10,000; 10,000–51,000; not applicable; and not reported. This classification was used for descriptive grouping purposes only and reflects the empirical distribution observed across the included studies, rather than predefined analytical thresholds. Most studies surveyed ≤1000 participants (62%). The ‘not applicable’ category was mainly associated with observational or secondary studies.
- Key feature 9—Study aims. Studies were classified according to their stated objectives as aiming to: (1) evaluate knowledge and/or behaviour or compare them with guidelines; (2) quantify or analyse specific food safety issues; or (3) contribute data supporting validated questionnaires. Overall, 52% of the documents fell into category 2, 41% into category 1, and 7% into category 3.
- Key feature 10—Study conclusions/discussion. Study conclusions and discussion sections were analysed (as reported in the studies) and classified using the same categories applied to study aims. The resulting distribution mirrored that observed for Key feature 9, indicating consistency between the stated objectives and reported conclusions.
- Key feature 11—Study result analysis. Study analyses were classified as narrative, descriptive statistics, statistical tests, or mixed. Statistical tests were reported in approximately 52% of the documents, reflecting considerable variability in analytical strategies.
- Key feature 12—Study administration. Study administration methods were classified as face-to-face or over-the-phone, online/postal/paper-based, or other. Face-to-face or over-the-phone administration was reported in approximately 51% of the documents, while around 42% employed online, postal, or paper-based approaches.
- Key feature 13—Geographical location. Studies conducted in high-income countries were classified by geographical region. Approximately 47% of the studies were conducted in Europe, followed by the United States (41%).
- Key feature 14—Food safety topics. The food safety topics addressed in the included studies were classified according to the number and type of topic areas covered, resulting in eight categories. Topic areas included microbiological, chemical, nutritional, home food safety practices (HFSP), and lifestyle-related aspects, addressed either individually or in combination. Studies were grouped into single-topic and mixed-topic categories, and mixed-topic studies were further classified according to whether two, three, or four topic areas were addressed. Single-topic studies represented the largest proportion of the included documents (45%), followed by studies addressing two food safety topics (38%). Studies covering three topic areas were less frequent (16%), while those addressing four topics accounted for a very small proportion of the corpus (1%). Notably, lifestyle-related aspects were not identified as a standalone topic but only in combination with other food safety topic areas. Detailed topic combinations and exact counts for the scientific and grey literature are reported in Supplementary Table S1. An aggregated overview of single- and mixed-topic studies is reported in Table 3, while detailed topic combinations and exact counts for the scientific and grey literature are provided in Supplementary Table S1.
- Key feature 15—Data collection period. Data collection periods were classified to describe the temporal distribution of the studies included in the review. Studies were grouped into five categories according to the period in which data were collected, including pre-2000 and a ‘not applicable’ (secondary studies)” category for studies (e.g., reviews or studies based on previously collected survey data) for which a specific data collection period could not be assigned. The largest proportion of the studies was conducted between 2011 and 2020, followed by 2001–2010. The pre-2000 and 2021–2023 periods were retained for descriptive completeness. The literature type breakdowns are reported in Table 3.

Note: Unless otherwise specified, percentages refer to the 274 included documents or are calculated within the relevant subgroup. Exact numbers for all categories are reported in Table 3.

## 4. Discussion

### 4.1. General

This scoping review mapped studies assessing consumers’ HFS-related KAP in high-income countries published during the period 2000–2023, synthesising 274 documents from the peer-reviewed and grey literature. Compared to earlier scoping and rapid reviews addressing related topics—such as the scoping review published in 2015 [19] and more recent rapid reviews focusing on specific populations [29]—the present study adopted a broader scope in terms of population coverage, time span, and document types. This approach enabled a detailed and up-to-date methodological overview of how HFS-related KAP studies are designed, implemented, and reported in high-income settings. To our knowledge, this is one of the most comprehensive mappings carried out in recent years in terms of temporal coverage and corpus size.

In accordance with the stated objectives, all review questions were addressed through the systematic identification, charting, and synthesis of study characteristics. This process led to the development of a 15-feature framework—which was refined and expanded from the eight features proposed in an earlier rapid review—describing how studies assessing HFS-related KAP are designed, implemented, and reported (Table 3). This framework provides a structured basis for interpreting recurring methodological patterns and areas of variability across studies.

**Figure 2:**
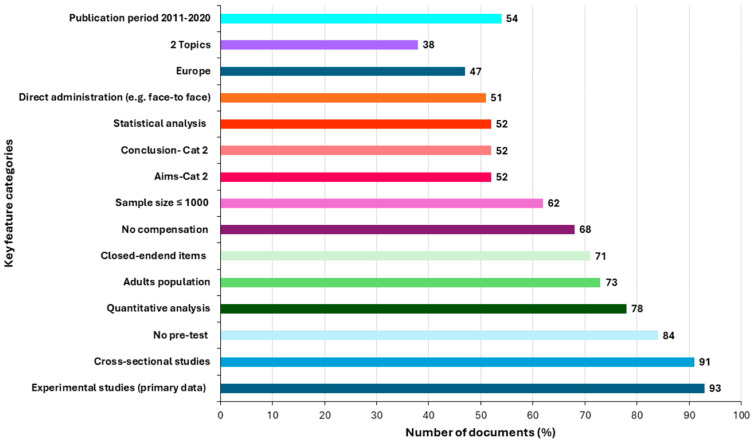
Key features based on data reported in Table 3. Histogram illustrating the percentage distribution of the 15 key features across the included documents. The x-axis represents the percentage of documents, while the y-axis reports the corresponding feature categories. Features are displayed according to the predefined numerical order of key features (1–15), as reported in Table 3.

To facilitate a synthetic interpretation of the overall distribution of the key features, an overall descriptive reading was adopted. As illustrated in Figure 2, key features showing a higher concentration of studies within a single category tended to display greater homogeneity, whereas features with a more even distribution across categories were associated with increased variability. When viewed through this descriptive lens, seven features appeared more consistently reported across studies, whereas eight showed greater heterogeneity. This distinction reflects an overall descriptive interpretation of the observed distributions rather than a strict categorisation of individual features. These two groups are discussed here as being indicative of a shared methodological core and of dimensions where key methodological and reporting characteristics remain more context-dependent or inconsistently reported, respectively.

### 4.2. More Homogeneous Key Features: Evidence of a Shared Methodological Core

The group of more homogeneous key features reveals a substantial degree of convergence in the overall design and analytical orientation of HFS-related KAP studies, suggesting the presence of a shared methodological core. Most studies relied on primary data collection rather than secondary analyses, reflecting a strong focus on generating original evidence on consumers’ HFS-related KAP. Cross-sectional designs predominated, consistent with the aim of capturing snapshots of practices and perceptions, while longitudinal or retrospective approaches were comparatively rare.

Another striking element of homogeneity is the widespread absence of pre- and post-intervention testing, indicating that studies were seldom used to evaluate learning outcomes or intervention effectiveness—echoing observations in earlier overviews of the adjacent literature [19]. This pattern highlights a methodological gap: while studies are extensively used to document HFS-related KAP, they are less frequently utilised to assess behavioural changes or intervention impact. The strong reliance on self-reported data, without testing components, may limit the ability of studies to guide evidence-based strategies for improving HFS. More broadly, it should be acknowledged that KAP-based approaches implicitly assume a linear relationship between knowledge, attitudes, and practices. This assumption has been extensively discussed as problematic in the literature, given the well-documented ‘knowledge–behaviour gap’. In the present review, the vast majority of studies relied on self-reported practices, while only a small number attempted to assess behaviour beyond self-reporting, for example, by means of observational components. Within this context, the near-total absence of pre- and post-intervention testing limits the ability of studies to capture behavioural changes or intervention impact over time.

From an analytical perspective, quantitative approaches based largely on closed-ended questions were the most frequently reported, facilitating scalable analysis and comparability across studies. On the other hand, this emphasis may also limit the exploration of contextual, cultural, or motivational factors influencing HFS behaviour. Participation was often voluntary and non-incentivised, a pragmatic choice that supports feasibility but can also introduce selection bias.

As regards target populations, most studies focused on adults, an operational category defined on the basis of author-reported classifications. In several cases, this category encompassed heterogeneous adult samples and, where no specific subgroup was explicitly identified, could also potentially include individuals meeting vulnerability criteria. A smaller proportion of studies explicitly targeted vulnerable populations or specific representative subgroups (e.g., students or defined community groups). This pragmatic categorisation supports synthesis across a diverse body of studies, while also highlighting areas where more clearly defined population-specific approaches could strengthen the comparability and interpretability of future research.

Taken together, these elements depict a shared methodological foundation underpinning most HFS-related KAP studies, characterised by a predominantly descriptive paradigm that has enabled extensive mapping of KAP-related outcomes. At the same time, they suggest potential areas for methodological evolution. For instance, where evaluation is intended, the incorporation of pre- and post-intervention or follow-up components could improve the assessment of changes. Additionally, in settings where behavioural drivers require deeper contextualisation, complementing quantitative analyses with qualitative components—i.e., adopting mixed-method approaches—might be considered.

### 4.3. Less Homogeneous Key Features: Variability Reflecting Complexity and Contextual Adaptation

The less homogeneous key features identify domains in which study practices diverged, often in relation to differing objectives, contexts, and operational constraints. This variability reflects the inherent complexity of research on HFS and the need to adapt study design and reporting practices to specific aims and settings.

Sample sizes varied considerably, with many studies conducted on a relatively small scale. While appropriate for exploratory or context-specific investigations, this variability can limit generalisation potential and complicate synthesis across studies. It should be noted that sample size was not used as a criterion for methodological quality assessment; rather, the observed dispersion reflects differences in study objectives, target populations, and feasibility considerations.

Study aims and conclusions also varied significantly. Some studies focused on quantifying specific FS issues, whereas others examined broader behavioural dimensions related to guidelines and recommendations. This diversity suggests that priorities across the literature remain heterogeneous. Analytical strategies ranged from narrative or descriptive approaches to the application of statistical tests, highlighting the absence of a single dominant framework for analysing HFS-related KAP evidence.

Operational and contextual choices added further variability. Modes of study administration and distribution varied between face-to-face or over-the-phone approaches and online, postal, or paper-based formats, reflecting both established practices and increasing digital uptake.

On a geographical level, most studies focused on Europe and the United States, with fewer studies identified in other high-income regions. This pattern was observed both in the scientific literature retrieved across multiple international databases and in the grey literature corpus. For the grey literature sources in particular, this distribution may also reflect language-related constraints that could have influenced the identification and analysis of governmental documents from some countries, in addition to differences in research activity and publication practices.

With respect to thematic scope, single-topic studies were slightly more common than multi-topic designs, and combinations of two topics were more common than broader integrations. Differences between the peer-reviewed and grey literature were also observed. Taken together, these factors may limit the capacity of individual studies to fully capture the multidimensional nature of HFS-related KAP. In this context, lifestyle-related aspects emerged as a particularly under-represented analytical dimension. Although lifestyle factors were occasionally addressed in combination with other topics—most frequently nutrition or microbiological risks—they were rarely considered as a standalone or overarching framework for interpreting HFS-related KAP. This contrasts with other areas of public health research, such as non-communicable disease prevention, where lifestyle constitutes a central analytical lens.

From a temporal perspective, research activity increased over time but remained unevenly distributed across periods characterised by evolving guidance and communication channels, which may complicate the interpretation of temporal trends. It should also be noted that publication year does not always coincide exactly with the data collection period. Several studies published in more recent years relied on data collected at an earlier time, reflecting the time-intensive nature of data analysis, interpretation, and manuscript preparation, and potentially resulting in a temporal gap between data collection and publication.

Importantly, the variability observed across these dimensions should not be interpreted as an indicator of methodological weakness. Rather, it points to areas where greater transparency in reporting, selective harmonisation of core methodological and reporting characteristics, and clearer documentation of study metadata may support the comparability and cumulative synthesis of evidence. At the same time, maintaining flexibility for context-specific adaptation remains essential in HFS research. In accordance with this, the variability described coexists with the shared methodological core identified in this review and reflects differences in study aims and settings rather than study conduct deficiencies.

## 5. Conclusions

This scoping review provides a comprehensive mapping of studies assessing consumers’ HFS-related KAP in high-income countries. By systematically characterising the design, implementation, and reporting of 274 studies published between 2000 and 2023, the review offers an integrated overview of how HFS-related KAP have been investigated across the scientific and grey literature sources.

Building on this synthesis, the review identified fifteen key features describing the recurring methodological and reporting characteristics of HFS-related KAP studies. This framework extends and refines the eight features proposed in an earlier rapid review and is intended as a descriptive tool to support transparency and comparability across future studies, rather than as a prescriptive model.

By making methodological and reporting choices more explicit, the framework may assist researchers, public health professionals, and institutions involved in food safety education and policy-related decision-making in contextualising findings, comparing evidence from population-based studies employing surveys, questionnaires, and interviews, and identifying areas where methodological and reporting practices could be improved.

A shared descriptive methodological paradigm was identified across the included literature, characterised by primary data collection, predominantly cross-sectional designs, quantitative analyses based on closed-ended instruments, and voluntary, non-incentivised participation. At the same time, substantial variability was observed with regard to study aims, sample size, analytical strategies, modes of administration, geographic coverage, thematic scope, and temporal distribution. As discussed, this variability reflects differences in research objectives and contexts rather than deficiencies in study conduct.

As regards thematic coverage, the distribution of food safety topics suggests a gradual shift from single-topic approaches towards more integrated frameworks combining microbiological aspects with HFSP and, increasingly, nutritional dimensions. By contrast, lifestyle-related aspects are not extensively integrated and are rarely considered as a standalone or overarching analytical framework, despite their potential relevance for understanding everyday food-related behaviour in domestic settings.

Overall, this review provides a structured and non-normative framework for supporting the design, reporting, and interpretation of future HFS-related KAP studies. By clarifying recurring methodological and reporting practices, highlighting areas of context-dependent variability, and identifying under-explored conceptual dimensions—such as the limited integration of lifestyle perspectives—the findings may contribute to the development of more transparent, comparable, and policy-relevant evidence to guide household FS education, communication, and intervention strategies.

## 6. Study Limitation

This scoping review has several limitations that should be considered when interpreting the findings. First, in keeping with scoping review methodology, no critical appraisal of methodological quality or risk of bias was undertaken. Consequently, the results should be interpreted as a descriptive mapping of methodological and reporting characteristics rather than an assessment of the strength or validity of the evidence.

Second, the operational categorisation of target populations—particularly the broad ‘adults’ category—was based on author-reported classifications and may have masked the presence of specific subgroups. This approach, while necessary to ensure synthesis across heterogeneous studies, may have limited the ability to draw more granular population-specific insights.

Third, although major bibliographic databases were systematically searched and the institutional and governmental grey literature was included, some relevant documents may have been overlooked. In particular, for the grey literature sources, language-related constraints may have influenced the coverage of documents published in languages not included in the search strategy. In addition, the exclusive focus on high-income countries limits the scope for generalising the findings to low- and medium-income settings, where household food safety challenges and research contexts may be substantially different.

Fourth, the analysis relied on the level of methodological detail reported in the included sources. Incomplete, inconsistent, or unclear reporting may have affected the classification of certain key features within the proposed framework, potentially underestimating variability in some methodological domains.

In addition, the review did not make a systematic distinction between self-reported and observational or objective measures of practices, as this aspect was not explicitly captured within the analytical framework. As a result, conclusions regarding how practices were measured should be interpreted at a conceptual rather than quantitative level.

Finally, this review includes studies published up to 3 August 2023. Extending the search beyond this date would have required repeating the screening and data extraction processes and was therefore beyond the scope of the present review. Future research could build on the present findings by extending the analysis to more recent publications in order to assess the consistency of emerging trends and methodological developments over time.

## Figures and Tables

**Table 1:** **PCC** **framework.**

**Population**	General population, including vulnerable people
**Concept**	Food safety, food handling, food literacy, health knowledge, surveys
**Context**	Households, high-income countries

**Table 2:** Eligibility criteria adopted for the selection of the studies.

Inclusion Criteria	Exclusion Criteria
Publications in English, Italian and Spanish. Studies involving the general population, including vulnerable people. International scope but focus on a high-income country setting. Focus only on home food safety and consumers’ behaviour, attitudes and knowledge. Primary peer-reviewed papers, including reviews and grey literature covering governmental and institutional reports only. Studies using observational/experimental research methods. Studies containing the terms ‘survey’, ‘interview’, ‘questionnaire’, and ‘cross-sectional’ in the title and/or abstract. Studies providing a description of the data collection methodology.	Studies focusing exclusively on patients or individuals with chronic diseases. Low- and middle-income countries. Studies focusing on ‘out of home’ food safety (e.g., restaurants, canteens, food trucks, hospitals) and on food business operators. Non-peer-reviewed records including monographs, letters, commentaries, dissertations, conference abstracts, books and articles merely expressing points of view/opinions, and other grey literature documents from governmental and institutional sources. Studies presenting only theoretical frameworks. Studies not containing the terms ‘survey’, ‘interview’, ‘questionnaire’, and ‘cross-sectional’ in the title and/or abstract. Studies not describing the data collection methodology.

**Table 3:** Key features and their associated options/values and relative descriptions.

Feature Number	Feature Name	Category	Total N° of Studies (274)	Scientific Literature (247)	Grey Literature (27)
1	Article type	Experimental	256	231	25
		Observational	15	13	2
		Other	3	3	0
2	Data collection method	Cross-sectional	250	225	25
		Longitudinal	10	10	0
		Retrospective	10	9	1
		Other	4	3	1
3	Testing	No	231	210	21
		Not applicable (observational)	19	16	3
		Yes	24	21	3
4	Data analysis method	Qualitative	26	18	8
		Quantitative	215	199	16
		Semi-quantitative	7	6	1
		Other (mixed)	26	24	2
5	Target population	Adults	200	180	20
		Representative sample group	20	17	3
		Vulnerable	54	50	4
6	Question Types	Closed questions, Likert, Pilot test	194	175	19
		Mixed	37	33	4
		Not reported	25	21	4
		Open questions and Pilot test	12	12	0
		Semi-closed questions	2	2	0
		Other	4	4	0
7	Cash/voucher compensation	Information not available	19	15	4
		No	186	172	14
		Other and Yes	69	60	9
8	Sample size	≤1000	171	168	3
		1000–10,000	76	60	16
		10,000–51,000	14	8	6
		Not applicable	12	10	2
		Not reported	1	1	0
9	Study aims	Cat. 1 *	113	100	13
		Cat. 2 *	143	135	8
		Cat. 3 *	18	12	6
10	Study conclusion/discussion	Cat. 1 *	113	100	13
		Cat. 2 *	143	135	8
		Cat. 3 *	18	12	6
11	Study result analysis	Descriptive Statistic	53	39	14
		Mixed	70	62	8
		Narrative	9	8	1
		Statistical	142	138	4
12	Study methods—administration/distribution	Face-to-face and Telephone	140	128	12
		Online, postal, paper	116	103	13
		Others (not reported or Observational)	18	16	2
13	Geographical location	America	113	107	6
		Asia	12	12	0
		Europe	130	110	20
		Oceania	12	11	1
		Mixed	7	7	0
14	Food safety topics	Mixed (2 topics)	104	91	13
		Mixed (3 topics)	44	41	3
		Mixed (4 topics)	4	4	0
		Single topic	123	112	11
15	Pubblication period	Pre-2000	24	23	1
		2001–2010	88	83	5
		2011–2020	150	131	19
		2021–2023	9	7	2
		Not applicable (observational) **	3	3	0

* Cat. 1 = studies aiming to evaluate knowledge and/or behaviour or compare them with guidelines. * Cat. 2 = studies aiming to quantify or analyse specific food safety issues. * Cat. 3 = studies aiming to contribute data supporting validated questionnaires. ** Not applicable (secondary studies)” indicates studies (e.g., reviews or studies based on previously collected survey data) for which a specific data collection period cannot be defined.

## Data Availability

The original contributions presented in this study are included in the article/Supplementary Materials. Further inquiries can be directed to the corresponding author.

## Supplementary Materials

The following supporting information can be downloaded at: https://www.mdpi.com/article/10.3390/foods15101730/s1, Supplementary Materials S1: PRISMA ScR Checklist; Supplementary Materials S2: Detailed search strategies for scientific database searches; Supplementary Materials S3: Detailed search strategies for governmental documents (grey literature); Supplementary Materials S4: Data extraction form; Table S1: Detailed distribution of food safety topic categories (key feature 14); Supplementary Materials S5: Full citation list of 274 relevant articles included in this scoping review.
