# Supplementary material for: Characterisation of Studies on Consumers’ Home Food Safety Knowledge, Attitudes, and Practices (KAP): A Scoping Review
Source: Foods. 2026 May 14;15(10):1730. doi: 10.3390/foods15101730 (PMC13205912; doi:10.3390/foods15101730)
Supplement: Supplementary file 1 [file foods-15-01730-s001.zip › foods-4234877-supplementary.pdf]

foods

MDPI

Characterisation of studies on consumers' home food safety knowledge, attitudes, and practices (KAP): a scoping review

Antonella Maugliani, Monica Valli, Francesca Maialetti, Francesca Baldi, Cinzia Civitareale, Manuela Luzi, Manlio Mammoli, Duilio Luca Bacocco, Donatella Gentili and Francesca De Battistis

Supplementary materials

Supplementary Materials S1: PRISMA-ScR Checklist

Supplementary Materials S2: Detailed search strategies for scientific database searches

Supplementary Materials S3: Detailed search strategies for governmental documents (grey literature)

Supplementary Materials S4: Data extraction form

Table S1

Supplementary Materials S5: Citation list of 274 relevant articles captured in this scoping review

5.1 Scientific literature (n = 247 papers)

5.2 Grey literature (n = 27 documents)

Supplementary Materials S1: **Preferred Reporting Items for Systematic reviews and Meta-Analyses extension for Scoping Reviews (PRISMA-ScR) Checklist**

SECTION	ITEM	PRISMA-ScR CHECKLIST ITEM	REPORTED ON PAGE #
**TITLE**			
Title	1	Identify the report as a scoping review.	Page 1
**ABSTRACT**			
Structured summary	2	Provide a structured summary that includes (as applicable): background, objectives, eligibility criteria, sources of evidence, charting methods, results, and conclusions that relate to the review questions and objectives.	Page 1
**INTRODUCTION**			
Rationale	3	Describe the rationale for the review in the context of what is already known. Explain why the review questions/objectives lend themselves to a scoping review approach.	Page 4
Objectives	4	Provide an explicit statement of the questions and objectives being addressed with reference to their key elements (e.g., population or participants, concepts, and context) or other relevant key elements used to conceptualize the review questions and/or objectives.	Page 4 and PCC framework page 5
**METHODS**			
Protocol and registration	5	Indicate whether a review protocol exists; state if and where it can be accessed (e.g., a Web address); and if available, provide registration information, including the registration number.	Page 5
Eligibility criteria	6	Specify characteristics of the sources of evidence used as eligibility criteria (e.g., years considered, language, and publication status), and provide a rationale.	Page 7 par.2.3
Information sources	7	Describe all information sources in the search (e.g., databases with dates of coverage and contact with authors to identify additional sources), as well as the date the most recent search was executed.	Pages 5;6 par. 2.2
Search	8	Present the full electronic search strategy for at least 1 database, including any limits used, such that it could be repeated.	Pages 5;6 par. 2.2
Selection of sources of evidence	9	State the process for selecting sources of evidence (i.e., screening and eligibility) included in the scoping review.	Page 8 par. 2.4
Data charting process	10	Describe the methods of charting data from the included sources of evidence (e.g., calibrated forms or forms that have been tested by the team before their use, and whether data charting was done independently or in duplicate) and any processes for obtaining and confirming data from investigators.	Page 8 par. 2.5
Data items	11	List and define all variables for which data were sought and any assumptions and simplifications made.	Page 8

SECTION	ITEM	PRISMA-ScR CHECKLIST ITEM	REPORTED ON PAGE #
Critical appraisal of individual sources of evidence§	12	If done, provide a rationale for conducting a critical appraisal of included sources of evidence; describe the methods used and how this information was used in any data synthesis (if appropriate).	Not done. Reasons Page 9
Synthesis of results	13	Describe the methods of handling and summarizing the data that were charted.	Pages 8;9 par. 2.6
**RESULTS**			
Selection of sources of evidence	14	Give numbers of sources of evidence screened, assessed for eligibility, and included in the review, with reasons for exclusions at each stage, ideally using a flow diagram.	Page 9
Characteristics of sources of evidence	15	For each source of evidence, present characteristics for which data were charted and provide the citations.	Page 10
Critical appraisal within sources of evidence	16	If done, present data on critical appraisal of included sources of evidence (see item 12).	Not done. Page 9
Results of individual sources of evidence	17	For each included source of evidence, present the relevant data that were charted that relate to the review questions and objectives.	Pages 10-13; par. 3.2
Synthesis of results	18	Summarize and/or present the charting results as they relate to the review questions and objectives.	Table_3; pages 13-14
**DISCUSSION**			
Summary of evidence	19	Summarize the main results (including an overview of concepts, themes, and types of evidence available), link to the review questions and objectives, and consider the relevance to key groups.	Page 15
Limitations	20	Discuss the limitations of the scoping review process.	Page 18
Conclusions	21	Provide a general interpretation of the results with respect to the review questions and objectives, as well as potential implications and/or next steps.	Page 17
**FUNDING**			
Funding	22	Describe sources of funding for the included sources of evidence, as well as sources of funding for the scoping review. Describe the role of the funders of the scoping review.	Page 18

JBI = Joanna Briggs Institute; PRISMA-ScR = Preferred Reporting Items for Systematic reviews and Meta-Analyses extension for Scoping Reviews. *From:* Tricco AC, Lillie E, Zarin W, O'Brien KK, Colquhoun H, Levac D, et al. PRISMA Extension for Scoping Reviews (PRISMAScR): Checklist and Explanation. Ann Intern Med. 2018;169:467–473. doi: 10.7326/M18-0850.

Supplementary Materials S2 **Detailed Search strategies for Scientific Database Searches**

**Date**	03/08/2023
**Database**	SCOPUS
**Search String**	(TITLE-ABS-KEY ("food safety" OR "food contamination*" OR "food microbiology" OR "food parasitology" OR "food adulteration*" OR "food adulterant" OR "foodborne" OR "food borne" OR "foodbourne" OR "food bourne" OR "food hygiene" OR "safe food" OR "food poisoning*" OR "food security" OR "food control" OR "food inspection*" OR "feed contamination*" OR "food contaminant*" OR "food pollution" OR "food pollutant*" OR "food spoilage" OR "food poisoning" OR "food infection*" OR "food intoxication*" OR "food toxicity" OR "food toxicology")) AND (TITLE-ABS-KEY ("Food Handling" OR "food processing*" OR "food packaging" OR "food container*" OR "Food Labelling" OR "Food Labeling" OR "Nutrition Labeling" OR "Nutrition Labelling" OR "food storage" OR "food shelf life" OR "food preservation" OR "food preparation*" OR "food practice*" OR "food washing" OR "Cooking" OR "cookery" OR "Refrigeration" OR "cold chain*" OR "Eating Utensil*" OR "cooking utensil*" OR "cutting board*" OR "sponge*" OR "towel*" OR "dishcloth*" OR "dish cloth*" OR "temperature*" OR "fast food*" OR "Convenience Food*" OR "Ready Prepared Food*" OR "Ready to Eat" OR "ready made meal*" OR "readymade meal*" OR "ready meal*" "ready-made meal*" OR "ready-made food*" OR "ready food*" OR "take away food*" OR "take out food*" OR "precooked food*" OR "pre cooked food*" OR "precooked meal*" OR "pre cooked meal*" OR "take away meal*" OR "takeaway meal*" OR "take out meal*" OR "takeout meal*" OR "take out food*" OR "takeout food*" OR "convenience meal*" OR "food packing" OR "food wrapping" OR "food conservation" OR "culinary preparation*" OR "foodstuffs preparation*" OR "meal* preparation*" OR "stored food" OR "consumer attitude*" OR "food*" OR "industrially prepared food*" OR "industrially prepared meal*" OR "ready-prepared meal*" OR "ready-to-eat food*" OR "ready-to-eat meal*" OR "ready-to-eat product*" OR "rte food*" OR "rte meal*")) AND (TITLE-ABS-KEY ("Risk factor*" OR "food risk*" OR pathogen* OR "chemical risk*" OR "cross contamina*" OR diet* OR perception OR "risk assessment")) AND (TITLE-ABS-KEY (housing OR home OR domestic OR household* OR kitchen* OR "home care" OR homecare)) AND (TITLE-ABS-KEY (person* OR "age groups" OR adolescent* OR adolescence OR teen* OR teenager* OR youth* OR adult* OR aged OR elderly OR "middle age" OR child OR children OR infant* OR population OR "oldest old" OR elder* OR "pregnant women" OR "pregnant woman" OR consumer* OR client* OR people OR individual* OR student* OR communit* OR famil* OR male* OR female* OR "age group" OR "very old" OR "young*" OR juvenile OR toddler* OR boy* OR girl* OR immunocompromised OR "Immuno compromised" OR immunocompromized OR "immuno compromized" OR immunosuppressed OR "Immuno suppressed" OR "compromised host" OR "compromized host" OR "compromised patient*" OR "compromized patient*" OR "immunecompromised" OR "immune compromised")) AND (TITLE-ABS-KEY (attitude* OR practice* OR knowledge OR aware* OR conscio* OR "food literacy" OR "health literacy" or AND behavior* OR behaviour*)) AND (TITLE-ABS-KEY (questionnaire* OR survey* OR interview*)) AND PUBYEAR > 1999 AND PUBYEAR < 2024

**Date**	03/08/2023
**Database**	PUBMED
**Search String**	((((EXP food safety [MH] OR food safety [tiab] OR food contamination* [tiab] OR "food microbiology"[tiab] OR "food parasitology"[tiab] OR food adulteration* [tiab] OR foodborne [tiab] OR food borne [tiab] OR foodbourne [tiab] OR food bourne [tiab] OR food hygiene [tiab] OR safe food [tiab] OR EXP foodborne diseases [MH] OR foodborne disease* [tiab] OR food borne disease* [tiab] OR food bourne disease* [tiab] OR foodborne illness* [tiab] OR food borne illness* [tiab] OR food bourne illness* [tiab] OR food poisoning* [tiab] OR food security [tiab]) AND (2000/1/1:3000/12/12[pdat]))) AND (((Food Handling [Mesh] OR food handling [tiab] OR food processing* [tiab] OR food packaging [MH] OR food packaging [tiab] OR food container* [tiab] OR Food Labelling [MH] OR Food Labelling [tiab] OR food labeling [tiab] OR Nutrition Labeling [tiab] OR nutrition labelling [tiab] OR food storage [MH] OR food storage [tiab] OR food shelf life [tiab] OR food preservation [MH] OR food preservation [tiab] OR food preparation* OR food practice* [tiab] OR food washing [tiab] OR Cooking[Mesh] OR cooking [tiab] OR cookery [tiab] OR Refrigeration [MH] OR Refrigeration [tiab] OR cold chain* [tiab] OR Cooking and Eating Utensils [MH] OR Eating Utensil* [tiab] OR cutting board* [tiab] OR sponge* [tiab] OR towel* [tiab] OR dishcloth* [tiab] OR dish cloth* [tiab] OR consumer behavior [MH] OR "consumer behavio*" OR temperature [MH] OR "temperature*" OR fast foods [MH] OR fast food* [tiab] OR Convenience Food* [tiab] OR Ready Prepared Food* [tiab] OR Ready to Eat [tiab] OR ready made meal* [tiab] OR readymade meal* [tiab] OR ready meal* [tiab] OR ready prepared meal* [tiab] OR ready food* [tiab] OR take away food* [tiab] OR take out food* [tiab] OR precooked food* [tiab] OR pre cooked food* [tiab] OR take away meal* [tiab] OR take out meal* [tiab] OR precooked meal* [tiab] OR pre cooked meal* [tiab] OR convenience meal* [tiab]) AND (2000/1/1:3000/12/12[pdat]))) AND (((Risk factors [MH] OR risk factor* [tiab] OR food risk* [tiab] OR pathogen* [tiab] OR chemical risk* [tiab] OR cross contamina* [tiab] OR "diet"[tiab] OR perception [MH] OR perception [tiab] OR risk perception [tiab]) AND (2000/1/1:3000/12/12[pdat]))) AND (((housing [MH] OR housing [tiab] OR home [tiab] OR domestic [tiab] OR household* [tiab] OR house hold* [tiab] OR kitchen* [tiab]) AND (2000/1/1:3000/12/12[pdat]))) AND (((persons [MH] OR person* [tiab] OR age groups [MH] OR adolescent [MH] OR Adolescent* [tiab] OR Adolescence [tiab] OR Teen* [tiab] OR Teenager* [tiab] OR Youth* [tiab] OR adult [MH] OR adult* [tiab] OR aged [MH] OR aged [tiab] OR elderly [tiab] OR middle aged [MH] OR middle aged [tiab] OR middle age [tiab] OR young adult [MH] OR young adult* [tiab] OR boy [tiab] OR boys [tiab] OR girl* [tiab] OR EXP child [MH] OR child [tiab] OR children [tiab] OR Preschool Child [tiab] OR Preschool Children [tiab] OR toddler* [tiab] OR EXP infant [MH] OR infant* [tiab] OR population [MH] OR population [tiab] OR EXP Aged, 80 and over [MH] OR oldest old [tiab] OR frail elderly [MH] OR elder* [tiab] OR pregnant women [MH] OR pregnant women [tiab] OR pregnant woman [tiab] OR consumer* [tiab] OR client* [tiab] OR people [tiab] OR individual* [tiab] OR student* [tiab] OR communit* [tiab] OR family [MH] OR famil* [tiab] OR Immunocompromised Host [MH] OR Immunocompromised [tiab] OR Immunosuppressed OR male [MH] OR male* [tiab] OR female [MH] OR female* [tiab]) AND (2000/1/1:3000/12/12[pdat]))) AND (((Health Knowledge, Attitudes, Practice [MH] OR Knowledge [tiab] OR attitude* [tiab] OR behavio* [tiab] OR practice* [tiab] OR aware* [tiab] OR conscio* [tiab] OR "food literacy" OR "health literacy") AND (2000/1/1:3000/12/12[pdat])))) AND (((Surveys and Questionnaires [MH] OR Survey* [tiab] OR Questionnaire* [tiab] OR interview* [tiab]) AND (2000/1/1:3000/12/12[pdat]))) - Saved search Filters: from 2000/1/1 - 3000/12/12

**Date**	03/08/2023
**Database**	EMBASE
**Search String**	('questionnaire'/exp OR 'questionnaire*':ab,kw,ti OR 'survey*':ab,kw,ti OR 'interview*':ab,kw,ti) AND ('food control'/exp OR 'food control' OR 'food control':ab,ti OR 'food inspection*':ab,ti OR 'food microbiology':ab,ti OR 'food parasitology':ab,ti OR 'food contamination'/exp OR 'food contamination' OR 'food contamination':ab,ti OR 'feed contamination':ab,ti OR 'food contaminant*':ab,ti OR 'food pollution':ab,ti OR 'food safety'/exp OR 'food safety' OR 'food safety':ab,ti OR 'food spoilage'/exp OR 'food spoilage' OR 'food spoilage':ab,ti OR 'food adulteration'/exp OR 'food adulteration' OR 'food adulteration*':ab,ti OR 'food poisoning'/exp OR 'food poisoning' OR 'food poisoning':ab,ti OR 'food borne disease*':ab,ti OR 'foodborne disease*':ab,ti OR 'foodbourne disease*':ab,ti OR 'food bourne disease*':ab,ti OR 'food borne illness*':ab,ti OR 'foodborne illness*':ab,ti OR 'foodbourne illness*':ab,ti OR 'food bourne illness*':ab,ti OR 'food infection*':ab,ti OR 'food intoxication*':ab,ti OR 'food toxicity':ab,ti OR 'food toxicology':ab,ti OR 'foodborne infection*':ab,ti OR 'foodbourne infection*':ab,ti OR 'food borne infection*':ab,ti OR 'food bourne infection*':ab,ti OR 'food hygiene'/exp OR 'food hygiene' OR 'food hygiene':ab,ti OR 'safe food*':ab,ti OR 'food security'/exp OR 'food security' OR 'food security':ab,ti) AND ('food handling'/de OR 'food handling':ab,ti OR 'food packaging'/de OR 'food packaging':ab,ti OR 'food labeling':ab,ti OR 'food labelling':ab,ti OR 'food packing':ab,ti OR 'food wrapping':ab,ti OR 'food preservation'/de OR 'food preservation':ab,ti OR 'food conservation':ab,ti OR 'food processing'/de OR 'food processing':ab,ti OR 'culinary preparation*':ab,ti OR 'food preparation*':ab,ti OR 'foodstuffs preparation*':ab,ti OR 'meal preparation*':ab,ti OR 'cooking'/de OR 'cooking':ab,ti OR 'cookery':ab,ti OR 'food washing'/de OR 'food washing':ab,ti OR 'food storage'/de OR 'food storage':ab,ti OR 'stored food':ab,ti OR 'cooking and eating utensils':ab,ti OR 'nutrition labeling'/de OR 'nutrition labeling':ab,ti OR 'food container*':ab,ti OR 'food shelf life':ab,ti OR 'food practice*':ab,ti OR 'refrigeration'/de OR 'refrigeration':ab,ti OR 'cold chain'/de OR 'cold chain':ab,ti OR 'cutting board*':ab,ti OR 'sponge'/exp OR 'sponge':ab,ti OR 'towel'/exp OR 'towel*':ab,ti OR 'dishcloth*':ab,ti OR 'dish cloth*':ab,ti OR 'consumer attitude'/exp OR 'consumer attitude*':ab,ti OR 'consumer behaviour*':ab,ti OR 'temperature'/de OR 'temperature':ab,ti OR 'food'/exp OR 'food*':ab,ti OR 'foodstuff*':ab,ti OR 'fast food*':ab,ti OR 'convenience food*':ab,ti OR 'convenience meal*':ab,ti OR 'industrially prepared food*':ab,ti OR 'ready meal*':ab,ti OR 'ready-made food*':ab,ti OR 'ready-made meal*':ab,ti OR 'ready-prepared food*':ab,ti OR 'ready-prepared meal*':ab,ti OR 'ready-to-eat food*':ab,ti OR 'ready-to-eat meal*':ab,ti OR 'ready-to-eat product*':ab,ti OR 'rte food*':ab,ti OR 'ready food*':ab,ti OR 'takeaway (food)'/exp OR 'takeaway food*':ab,ti OR 'take away food*':ab,ti OR 'takeaway meal*':ab,ti OR 'take away meal*':ab,ti OR 'take out food*':ab,ti OR 'takeout meal*':ab,ti OR 'take out meal*':ab,ti OR 'takeout food*':ab,ti OR 'precooked food*':ab,ti OR 'pre cooked food*':ab,ti OR 'precooked meal*':ab,ti OR 'pre cooked meal*':ab,ti) AND ('risk factor'/exp OR 'risk factor*':ab,ti,kw OR 'food risk*':ab,ti,kw OR pathogen*:ab,ti,kw OR 'chemical risk*':ab,ti,kw OR 'cross contamination'/exp OR 'cross contamination':ab,ti,kw OR diet:ab,ti OR 'perception'/de OR 'perception':ab,kw,ti OR 'risk perception'/exp OR 'risk perception':ab,kw,ti OR 'risk assessment'/de OR 'risk assessment':ab,kw,ti) AND ('home'/exp OR 'home':ab,kw,ti OR 'home care'/de OR 'home care':kw,ab,ti OR 'kitchen'/exp OR 'kitchen*':ab,ti,kw OR 'domestic':kw,ab,ti OR 'household'/exp OR 'household':ab,ti,kw OR 'house hold':kw,ab,ti OR 'housing'/exp OR 'housing':ab,ti) AND ('groups by age'/de OR 'groups by age':ab,ti OR 'adult'/de OR 'adult*':ab,ti OR 'aged'/exp OR 'aged':ab,ti OR 'aged people':ab,ti OR 'aged person*':ab,ti OR 'elderly people':ab,ti OR 'elderly person*':ab,ti OR 'aged, 80 and over':ab,ti OR 'very old':ab,ti OR elderly:ab,ti OR 'middle aged'/exp OR 'middle aged':ab,ti OR 'middle age':ab,ti OR 'young adult'/exp OR 'young adult':ab,ti OR 'young*':ab,ti OR 'prime adult*':ab,ti OR 'juvenile'/de OR 'juvenile':ab,ti OR youth*:ab,ti OR 'adolescent'/de OR adolescent*:ab,ti OR adolescence:ab,ti OR teen*:ab,ti OR teenager*:ab,ti OR 'child'/de OR 'child':ab,ti OR children:ab,ti OR 'preschool child'/exp OR 'preschool child':ab,ti OR

	'pre school child':ab,ti OR 'preschool children':ab,ti OR 'school child'/exp OR 'school child':ab,ti OR 'school children':ab,ti OR 'toddler'/exp OR 'toddler*':ab,ti OR 'boy'/exp OR 'boy*':ab,ti OR 'girl'/exp OR 'girl*':ab,ti OR 'infant'/exp OR 'infant*':ab,ti OR 'population'/exp OR 'population':ab,ti OR 'pregnant woman'/exp OR 'pregnant woman':ab,ti OR 'pregnant women':ab,ti OR 'consumer'/exp OR 'consumer':ab,ti OR 'clients'/exp OR 'client*':ab,ti OR people:ab,ti OR 'individual*':ab,ti OR 'student'/exp OR 'student*':ab,ti OR 'communit*':ab,ti OR 'family'/de OR 'famil*':ti,ab OR 'immunocompromised patient'/exp OR 'immunocompromised patient':ab,ti OR 'compromised host':ab,ti OR 'compromized host':ab,ti OR 'immune compromised host':ab,ti OR 'immune compromised patient*':ab,ti OR 'immunocompromised host':ab,ti OR 'immunocompromized host':ab,ti OR 'immunocompromized patient*':ab,ti OR 'male'/de OR 'male*':ab,ti OR 'female'/de OR 'female*':ab,ti OR 'woman':ab,ti OR 'women':ab,ti) AND ('attitude to health'/exp OR 'attitude to health':ab,kw,ti OR knowledge:ab,kw,ti OR 'behavio*':ab,ti OR 'practice*':ab,ti OR 'aware*':ab,ti OR 'conscio*':ab,ti OR 'food literacy':ab,kw,ti OR 'health literacy'/exp OR 'health literacy':ab,ti,kw) AND (2000:py OR 2001:py OR 2002:py OR 2003:py OR 2004:py OR 2005:py OR 2006:py OR 2007:py OR 2008:py OR 2009:py OR 2010:py OR 2011:py OR 2012:py OR 2013:py OR 2014:py OR 2015:py OR 2016:py OR 2017:py OR 2018:py OR 2019:py OR 2020:py OR 2021:py OR 2022:py)

**Date**	03/08/2023
**Database**	Web of Science
**Search String**	(((((((TS=("food safety" OR "food contamination*" OR "food microbiology" OR "food parasitology" OR "food adulteration*" OR "food adulterant" OR "foodborne" OR "food borne" OR "foodbourne" OR "food bourne" OR "food hygiene" OR "safe food" OR "food poisoning*" OR "food security" OR "food control" OR "food inspection*" OR "feed contamination*" OR "food contaminant*" OR "food pollution" OR "food pollutant*" OR "food spoilage" OR "food poisoning" OR "food infection*" OR "food intoxication*" OR "food toxicity" OR "food toxicology")) AND TS=("Food Handling" OR "food processing*" OR "food packaging" OR "food container*" OR "Food Labelling" OR "Food Labeling" OR "Nutrition Labeling" OR "food storage" OR "food shelf life" OR "food preservation" OR "food preparation*" OR "food practice*" OR "food washing" OR "Cooking" OR "cookery" OR "Refrigeration" OR "cold chain*" OR "Eating Utensil*" OR "cooking utensil*" OR "cutting board*" OR "sponge*" OR "towel*" OR "dishcloth*" OR "dish cloth*" OR "temperature*" OR "fast food*" OR "Convenience Food*" OR "Ready Prepared Food*" OR "Ready to Eat" OR "ready made meal*" OR "readymade meal*" OR "ready meal*" "ready-made meal*" OR "ready-made food*" OR "ready food*" OR "take away food*" OR "take out food*" OR "precooked food*" OR "pre cooked food*" OR "precooked meal*" OR "pre cooked meal*" OR "take away meal*" OR "takeaway meal*" OR "take out meal*" OR "takeout meal*" OR "take out food*" OR "takeout food*" OR "convenience meal*" OR "food packing" OR "food wrapping" OR "food conservation" OR "culinary preparation*" OR "foodstuffs preparation*" OR "meal* preparation*" OR "stored food" OR "consumer attitude*" OR "food*" OR "industrially prepared food*" OR "industrially prepared meal*" OR "ready-prepared meal*" OR "ready-to-eat food*" OR "ready-to-eat meal*" OR "ready-to-eat product*" OR "rte food*" OR "rte meal*”)) AND TS=("Risk factor*" OR "food risk*" OR pathogen* OR "chemical risk*" OR "cross contamina*" OR diet* OR perception OR "risk assessment")) AND TS=(housing OR home OR domestic OR household* OR kitchen* OR "home care" OR homecare)) AND TS=(person* OR "age groups" OR Adolescent* OR Adolescence OR Teen* OR Teenager* OR Youth* OR adult* OR aged OR elderly OR "middle age" OR child OR children OR infant* OR population OR "oldest old" OR elder* OR "pregnant women" OR "pregnant woman" OR consumer* OR client* OR people OR individual* OR student* OR communit* OR famil* OR male* OR female* OR "age group" OR "very old" OR "young*" OR juvenile OR toddler* OR boy* OR girl* OR Immunocompromised OR "Immuno compromised" OR immunocompromized OR "immuno compromized" OR Immunosuppressed OR "Immuno suppressed" OR "compromised host" OR "compromized host" OR "compromised patient*" OR "compromized patient*" OR "immunecompromised" OR "immune compromised")) AND TS=(Attitude* OR Practice* OR Knowledge OR aware* OR conscio* OR "food literacy" OR "health literacy" OR behavior* OR behaviour*))AND TS=(questionnaire* OR survey* OR interview*))<br>Timespan: 2000-01-01 to 2023-12-31 (Publication Date)

**Date**	03/08/2023
**Database**	COCHRANE

Search String

ID	Search
#1	MeSH descriptor: [Food Safety] explode all trees
#2	MeSH descriptor: [Foodborne Diseases] explode all trees
#3	food safety:ti,ab OR (food NEXT contamination*):ti,ab OR "food microbiology":ti,ab OR "food parasitology":ti,ab OR "food adulteration*":ti,ab OR foodborne:ti,ab OR "food borne":ti,ab OR foodbourne:ti,ab OR "food bourne":ti,ab OR "food hygiene":ti,ab OR "safe food":ti,ab OR "food control":ti,ab OR (food NEXT inspection*):ti,ab OR (feed NEXT contamination*):ti,ab OR (food NEXT inspection*):ti,ab OR (food NEXT contaminant*):ti,ab OR "food pollution":ti,ab OR (food NEXT poisoning*):ti,ab OR "food security":ti,ab OR "food control":ti,ab OR (food NEXT inspection*):ti,ab OR (feed NEXT contamination*):ti,ab OR (food NEXT inspection*):ti,ab OR (food NEXT contaminant*):ti,ab OR "food pollution":ti,ab OR (food NEXT pollutant*):ti,ab OR "food spoilage":ti,ab OR (food NEXT infection*):ti,ab OR (food NEXT intoxication*):ti,ab OR "food toxicity":ti,ab OR "food toxicology":ti,ab (Word variations have been searched)
#4	#1 OR #2 OR #3
#5	MeSH descriptor: [Food Packaging] this term only
#6	MeSH descriptor: [Food Labeling] this term only
#7	MeSH descriptor: [Food Storage] this term only
#8	MeSH descriptor: [Cooking] explode all trees
#9	MeSH descriptor: [Cooking and Eating Utensils] this term only
#10	MeSH descriptor: [Refrigeration] this term only
#11	MeSH descriptor: [Consumer Behavior] this term only
#12	MeSH descriptor: [Temperature] this term only
#13	MeSH descriptor: [Fast Foods] this term only
#14	MeSH descriptor: [Food Handling] this term only
#15	MeSH descriptor: [Food Preservation] explode all trees
#16	Food Handling:ti,ab OR (food NEXT processing*):ti,ab OR "food packaging":ti,ab OR (food NEXT container*):ti,ab OR "Food Labelling":ti,ab OR "Food Labeling":ti,ab OR "Nutrition Labelling":ti,ab OR "Nutrition Labeling":ti,ab OR "food storage":ti,ab OR "food shelf life":ti,ab OR "food preservation":ti,ab OR (food NEXT preparation*):ti,ab OR (food NEXT practice*):ti,ab OR "food washing":ti,ab OR "Cooking":ti,ab OR "cookery":ti,ab OR "Refrigeration":ti,ab OR (cold NEXT chain*):ti,ab OR (Eating NEXT Utensil*):ti,ab OR (cooking NEXT utensil*):ti,ab OR (cutting NEXT board*):ti,ab OR sponge*:ti,ab OR towel*:ti,ab OR dishcloth*:ti,ab OR (dish NEXT cloth*):ti,ab OR "temperature*":ti,ab OR (fast NEXT food*):ti,ab OR (Convenience NEXT Food*):ti,ab OR (Ready NEXT Prepared NEXT Food*):ti,ab OR "Ready to Eat":ti,ab OR (ready NEXT made NEXT meal*):ti,ab OR (readymade NEXT meal*):ti,ab OR (ready NEXT meal*):ti,ab OR (ready-made NEXT meal*):ti,ab OR (ready-made NEXT food*):ti,ab OR (ready NEXT food*):ti,ab OR (take NEXT away NEXT food*):ti,ab OR (take NEXT out NEXT food*):ti,ab OR (precooked NEXT food*):ti,ab OR (pre NEXT cooked NEXT food*):ti,ab OR (precooked NEXT meal*):ti,ab OR (pre NEXT

			cooked NEXT meal*):ti,ab OR (take NEXT away NEXT meal*):ti,ab OR (takeaway NEXT meal*):ti,ab OR (take NEXT out NEXT meal*):ti,ab OR (takeout NEXT meal*):ti,ab OR (take NEXT out NEXT food*):ti,ab OR (takeout NEXT food*):ti,ab OR (convenience NEXT meal*):ti,ab OR "food packing":ti,ab OR "food wrapping":ti,ab OR "food conservation":ti,ab OR (culinary NEXT preparation*):ti,ab OR (foodstuffs NEXT preparation*):ti,ab OR (meal* NEXT preparation*):ti,ab OR "stored food":ti,ab OR (consumer NEXT behavio*):ti,ab OR (consumer NEXT attitude*):ti,ab OR "food*":ti,ab OR (industrially NEXT prepared NEXT food*):ti,ab OR (industrially NEXT prepared NEXT meal*):ti,ab OR (ready-prepared NEXT meal*):ti,ab OR (ready-to-eat NEXT food*):ti,ab OR (ready-to-eat NEXT meal*):ti,ab OR (ready-to-eat NEXT product*):ti,ab OR (rte NEXT food*):ti,ab OR (rte NEXT meal*):ti,ab
		#17	#5 OR #6 OR #7 OR #8 OR #9 OR #10 OR #11 OR #12 OR #13 OR #14 OR #15 OR #16 (Word variations have been searched)
		#18	MeSH descriptor: [Risk Factors] this term only
		#19	MeSH descriptor: [Perception] this term only
		#20	(Risk NEXT factor*):ti,ab OR food:ti,ab OR (food NEXT risk*):ti,ab OR pathogen*:ti,ab OR (chemical NEXT risk*):ti,ab OR (cross NEXT contamina*):ti,ab OR diet*:ti,ab OR perception:ti,ab OR (risk NEXT assessment):ti,ab (Word variations have been searched)
		#21	#18 OR #19 OR #20 (Word variations have been searched)
		#22	MeSH descriptor: [Housing] this term only
		#23	housing:ti,ab OR home:ti,ab OR domestic:ti,ab OR household*:ti,ab OR (house NEXT hold*):ti,ab OR kitchen*:ti,ab OR homecare:ti,ab (Word variations have been searched)
		#24	#22 OR #23
		#25	MeSH descriptor: [undefined] explode all trees
		#26	MeSH descriptor: [Age Groups] this term only
		#27	MeSH descriptor: [Adolescent] this term only
		#28	MeSH descriptor: [Adult] this term only
		#29	MeSH descriptor: [Aged] explode all trees
		#30	MeSH descriptor: [Middle Aged] this term only
		#31	MeSH descriptor: [Young Adult] this term only
		#32	MeSH descriptor: [Child] explode all trees
		#33	MeSH descriptor: [Infant] explode all trees
		#34	MeSH descriptor: [Population] this term only
		#35	MeSH descriptor: [Aged, 80 and over] explode all trees
		#36	MeSH descriptor: [Frail Elderly] this term only
		#37	MeSH descriptor: [Pregnant Women] this term only
		#38	MeSH descriptor: [Family] this term only
		#39	MeSH descriptor: [Immunocompromised Host] this term only

#40	MeSH descriptor: [Male] explode all trees
#41	MeSH descriptor: [Female] explode all trees
#42	person*:ti,ab OR "age groups":ti,ab OR Adolescent*:ti,ab OR Adolescence:ti,ab OR Teen*:ti,ab OR Teenager*:ti,ab OR Youth*:ti,ab OR adult*:ti,ab OR aged:ti,ab OR elderly:ti,ab OR "middle age":ti,ab OR child:ti,ab OR children:ti,ab OR infant*:ti,ab OR population:ti,ab OR "oldest old":ti,ab OR elder*:ti,ab OR "pregnant women":ti,ab OR "pregnant woman":ti,ab OR consumer*:ti,ab OR client*:ti,ab OR people:ti,ab OR individual*:ti,ab OR student*:ti,ab OR communit*:ti,ab OR famil*:ti,ab OR male*:ti,ab OR female*:ti,ab OR "age group":ti,ab OR "very old":ti,ab OR "young*":ti,ab OR juvenile OR toddler* OR boy* OR girl* OR Immunocompromised OR "Immuno compromised" OR immunocompromized OR "immuno compromized" OR Immunosuppressed OR "Immuno suppressed":ti,ab OR "compromised host":ti,ab OR "compromized host":ti,ab OR (compromised NEXT patient*):ti,ab OR (compromized NEXT patient*):ti,ab OR "immunecompromised":ti,ab OR "immune compromised":ti,ab (Word variations have been searched)
#43	#25 OR #26 OR #27 OR #28 OR #29 OR #30 OR #31 OR #32 OR #33 OR #34 OR #35 OR #36 OR #37 OR #38 OR # 38 OR #40 OR #41 OR #42
#44	MeSH descriptor: [Health Knowledge, Attitudes, Practice] this term only
#45	Attitude*:ti,ab OR Practice*:ti,ab OR Knowledge:ti,ab OR aware*:ti,ab OR conscio*:ti,ab OR "food literacy":ti,ab OR "health literacy":ti,ab OR behavior*:ti,ab OR behaviour*:ti,ab (Word variations have been searched)
#46	#44 OR #45 (Word variations have been searched)
#47	#4 AND #17 AND #21 AND #24 AND #43 AND #46 (Word variations have been searched)
#48	MeSH descriptor: [Surveys and Questionnaires] explode all trees
#49	questionnaire*:ti,ab OR survey*:ti,ab OR interview*:ti,ab
#50	#48 OR #49
#51	#47 AND #50
#52	MeSH descriptor: [Food Safety] explode all trees
#53	MeSH descriptor: [Foodborne Diseases] explode all trees
#54	food safety:ti,ab OR (food NEXT contamination*):ti,ab OR "food microbiology":ti,ab OR "food parasitology":ti,ab OR "food adulteration*":ti,ab OR foodborne:ti,ab OR "food borne":ti,ab OR foodbourne:ti,ab OR "food bourne":ti,ab OR "food hygiene":ti,ab OR "safe food":ti,ab OR (food NEXT poisoning*):ti,ab OR "food security":ti,ab OR "food control":ti,ab OR (food NEXT inspection*):ti,ab OR (feed NEXT contamination*):ti,ab OR (food NEXT inspection*):ti,ab OR (food NEXT contaminant*):ti,ab OR "food pollution":ti,ab OR (food NEXT pollutant*):ti,ab OR "food spoilage":ti,ab OR (food NEXT infection*):ti,ab OR (food NEXT intoxication*):ti,ab OR "food toxicity":ti,ab OR "food toxicology":ti,ab (Word variations have been searched)
#55	#52 OR #53 OR #54
#56	MeSH descriptor: [Food Packaging] this term only
#57	MeSH descriptor: [Food Labeling] this term only
#58	MeSH descriptor: [Food Storage] this term only
#59	MeSH descriptor: [Cooking] explode all trees

	#60	MeSH descriptor: [Cooking and Eating Utensils] this term only
	#61	MeSH descriptor: [Refrigeration] this term only
	#62	MeSH descriptor: [Consumer Behavior] this term only
	#63	MeSH descriptor: [Temperature] this term only
	#64	MeSH descriptor: [Fast Foods] this term only
	#65	MeSH descriptor: [Food Handling] this term only
	#66	MeSH descriptor: [Food Preservation] explode all trees
	#67	Food Handling:ti,ab OR (food NEXT processing*):ti,ab OR "food packaging":ti,ab OR (food NEXT container*):ti,ab OR "Food Labelling":ti,ab OR "Food Labeling":ti,ab OR "Nutrition Labelling":ti,ab OR "Nutrition Labeling":ti,ab OR "food storage":ti,ab OR "food shelf life":ti,ab OR "food preservation":ti,ab OR (food NEXT preparation*):ti,ab OR (food NEXT practice*):ti,ab OR "food washing":ti,ab OR "Cooking":ti,ab OR "cookery":ti,ab OR "Refrigeration":ti,ab OR (cold NEXT chain*):ti,ab OR (Eating NEXT Utensil*):ti,ab OR (cooking NEXT utensil*):ti,ab OR (cutting NEXT board*):ti,ab OR sponge*:ti,ab OR towel*:ti,ab OR dishcloth*:ti,ab OR (dish NEXT cloth*):ti,ab OR "temperature*":ti,ab OR (fast NEXT food*):ti,ab OR (Convenience NEXT Food*):ti,ab OR (Ready NEXT Prepared NEXT Food*):ti,ab OR "Ready to Eat":ti,ab OR (ready NEXT made NEXT meal*):ti,ab OR (readymade NEXT meal*):ti,ab OR (ready NEXT meal*):ti,ab OR (ready-made NEXT meal*):ti,ab OR (ready-made NEXT food*):ti,ab OR (ready NEXT food*):ti,ab OR (take NEXT away NEXT food*):ti,ab OR (take NEXT out NEXT food*):ti,ab OR (precooked NEXT food*):ti,ab OR (pre NEXT cooked NEXT food*):ti,ab OR (precooked NEXT meal*):ti,ab OR (pre NEXT cooked NEXT meal*):ti,ab OR (take NEXT away NEXT meal*):ti,ab OR (takeaway NEXT meal*):ti,ab OR (take NEXT out NEXT meal*):ti,ab OR (takeout NEXT meal*):ti,ab OR (take NEXT out NEXT food*):ti,ab OR (takeout NEXT food*):ti,ab OR (convenience NEXT meal*):ti,ab OR "food packing":ti,ab OR "food wrapping":ti,ab OR "food conservation":ti,ab OR (culinary NEXT preparation*):ti,ab OR (foodstuffs NEXT preparation*):ti,ab OR (meal* NEXT preparation*):ti,ab OR "stored food":ti,ab OR (consumer NEXT behavio*):ti,ab OR (consumer NEXT attitude*):ti,ab OR "food*":ti,ab OR (industrially NEXT prepared NEXT food*):ti,ab OR (industrially NEXT prepared NEXT meal*):ti,ab OR (ready-prepared NEXT meal*):ti,ab OR (ready-to-eat NEXT food*):ti,ab OR (ready-to-eat NEXT meal*):ti,ab OR (ready-to-eat NEXT product*):ti,ab OR (rte NEXT food*):ti,ab OR (rte NEXT meal*):ti,ab
	#68	#56 OR #57 OR #58 OR #59 OR #60 OR #61 OR #62 OR #63 OR #64 OR #65 OR #66 OR #67 (Word variations have been searched)
	#69	MeSH descriptor: [Risk Factors] this term only
	#70	MeSH descriptor: [Perception] this term only
	#71	(Risk NEXT factor*):ti,ab OR food:ti,ab OR (food NEXT risk*):ti,ab OR pathogen*:ti,ab OR (chemical NEXT risk*):ti,ab OR (cross NEXT contamina*):ti,ab OR diet*:ti,ab OR perception:ti,ab OR (risk NEXT assessment):ti,ab (Word variations have been searched)
	#72	#69 OR #70 OR #71 (Word variations have been searched)
	#73	MeSH descriptor: [Housing] this term only
	#74	housing:ti,ab OR home:ti,ab OR domestic:ti,ab OR household*:ti,ab OR (house NEXT hold*):ti,ab OR kitchen*:ti,ab OR homecare:ti,ab (Word variations have been searched)

#75	#73 OR #74
#76	MeSH descriptor: [undefined] explode all trees
#77	MeSH descriptor: [Age Groups] this term only
#78	MeSH descriptor: [Adolescent] this term only
#79	MeSH descriptor: [Adult] this term only
#80	MeSH descriptor: [Aged] explode all trees
#81	MeSH descriptor: [Middle Aged] this term only
#82	MeSH descriptor: [Young Adult] this term only
#83	MeSH descriptor: [Child] explode all trees
#84	MeSH descriptor: [Infant] explode all trees
#85	MeSH descriptor: [Population] this term only
#86	MeSH descriptor: [Aged, 80 and over] explode all trees
#87	MeSH descriptor: [Frail Elderly] this term only
#88	MeSH descriptor: [Pregnant Women] this term only
#89	MeSH descriptor: [Family] this term only
#90	MeSH descriptor: [Immunocompromised Host] this term only
#91	MeSH descriptor: [Male] explode all trees
#92	MeSH descriptor: [Female] explode all trees
#93	person*:ti,ab OR "age groups":ti,ab OR Adolescent*:ti,ab OR Adolescence:ti,ab OR Teen*:ti,ab OR Teenager*:ti,ab OR Youth*:ti,ab OR adult*:ti,ab OR aged:ti,ab OR elderly:ti,ab OR "middle age":ti,ab OR child:ti,ab OR children:ti,ab OR infant*:ti,ab OR population:ti,ab OR "oldest old":ti,ab OR elder*:ti,ab OR "pregnant women":ti,ab OR "pregnant woman":ti,ab OR consumer*:ti,ab OR client*:ti,ab OR people:ti,ab OR individual*:ti,ab OR student*:ti,ab OR communit*:ti,ab OR famil*:ti,ab OR male*:ti,ab OR female*:ti,ab OR "age group":ti,ab OR "very old":ti,ab OR "young*":ti,ab OR juvenile OR toddler* OR boy* OR girl* OR Immunocompromised OR "Immuno compromised" OR immunocompromized OR "immuno compromized" OR Immunosuppressed OR "Immuno suppressed":ti,ab OR "compromised host":ti,ab OR "compromized host":ti,ab OR (compromised NEXT patient*):ti,ab OR (compromized NEXT patient*):ti,ab OR "immunecompromised":ti,ab OR "immune compromised":ti,ab (Word variations have been searched)
#94	#76 OR #77 OR #78 OR #79 OR #80 OR #81 OR #82 OR #83 OR #84 OR #85 OR #86 OR #87 OR #88 OR #89 OR # 38 OR #91 OR #92 OR #93
#95	MeSH descriptor: [Health Knowledge, Attitudes, Practice] this term only
#96	Attitude*:ti,ab OR Practice*:ti,ab OR Knowledge:ti,ab OR aware*:ti,ab OR conscio*:ti,ab OR "food literacy":ti,ab OR "health literacy":ti,ab OR behavior*:ti,ab OR behaviour*:ti,ab (Word variations have been searched)
#97	#95 OR #96 (Word variations have been searched)
#98	#55 AND #68 AND #72 AND #75 AND #94 AND #97 (Word variations have been searched)
#99	MeSH descriptor: [Surveys and Questionnaires] explode all trees

		#100	questionnaire*:ti,ab OR survey*:ti,ab OR interview*:ti,ab	
		#101	#99 OR #100	
		#102	#98 AND #101	

Supplementary Materials S3 **Detailed Search strategies for Governmental documents (grey literature)**

- **Common full core search strings used for retrieving information:**

A. English Search Query (ESQ): ((questionnaire OR survey) AND ("food risk" OR "food safety" OR "food consumption") AND (consumer OR human OR citizen) AND (home OR domestic) AND ("food handling" OR "food preparation" OR "food contamination") AND (-restaurant))

B. Italian Search Query (ISQ) used for Italy country: ((questionario OR sondaggio) AND ("rischio alimentare" OR "sicurezza alimentare" OR "consumo alimentare") AND (consumatore OR essere umano OR cittadino) AND (casa OR domestico) AND ("manipolazione degli alimenti" OR "preparazione degli alimenti" OR " contaminazione alimentare") AND (-ristorante))

C. Spanish Search Query (SSQ) used for Spain/Chile country: ((cuestionario OR encuesta) AND ("riesgo alimentario" OR "seguridad alimentaria" OR "consumo de alimentos") AND (consumidor OR humano OR ciudadano) AND (hogar OR doméstico) AND ("manipulación de alimentos" OR "preparación de alimentos" OR " contaminación de alimentos") AND (-restaurante))
D. Data of research: 17 October 2023

- **Table with details for each Countries:**

EUROPEAN COUNTRIES analysed with RESULTS<br>TOT N° Countries: 11<br>TOT N° Documents: 52			
**Search String**	ESQ		
**COUNTRY**	FINLAND		
**Institutional Name**	Finnish Food Authority.<br>Ministry of Agriculture and Forestry.	**Web Site included in the Search string**	site:ruokavirasto.fi<br>site:stm.fi<br>site:mmm.fi
**Documents**	https://www.ruokavirasto.fi/globalassets/tietoa-meista/julkaisut/julkaisusarjat/tutkimukset/riskiraportit/broilersalmo_5.pdf		
**COUNTRY**	FRANCE		
**Institutional Name**	Agency for Food, Environmental and Occupational Health & Safety.<br>Ministry of Solidarity and Health.<br>Ministry of Agriculture and Food.	**Web Site included in the Search string**	site:anses.fr<br>site:sante.gouv.fr<br>site:agriculture.gouv.fr
**Documents**	https://www.anses.fr/en/system/files/BIORISK2016SA0183RaEN.pdf<br>https://www.anses.fr/en/system/files/MIC-Ra-campylobacter.pdf		
**COUNTRY**	GREECE		
**Institutional Name**	Hellenic Food Authority.<br>National Public.<br>Health Organization.<br>Ministry of Rural Development and Food.	**Web Site included in the Search string**	site:efet.gr<br>site:eody.gov.gr<br>site:minagric.gr
**Documents**	https://www.efet.gr/files/JRC128410_01.pdf		
**COUNTRY**	GERMANY		
**Institutional Name**	Federal Institute for Risk Assessment.<br>Federal Ministry of Food and Agriculture.<br>Federal Research Institute of Nutrition and Food.	**Web Site included in the Search string**	site:bfr.bund.de<br>site:bmel.de<br>site:mri.bund.de
**Documents**	https://mobil.bfr.bund.de/cm/343/risk_issues_executive_summary_on_food_safety.pdf		

**COUNTRY**	NETHERLANDS		
**Institutional Name**	RIVM.<br>Wageningen University & Research.<br>Food and Consumer Products Safety Authority<br>Dutch Ministry of Agriculture, Nature and Food Quality- LNV	**Web Site included in the Search string**	site:rivm.nl<br>ite:wur.nl<br>site:nvwa.nl<br>site:dcnanature.org/lnv
**Documents**	https://www.rivm.nl/bibliotheek/rapporten/2016-0196.pdf<br>https://www.rivm.nl/en/food-safety<br>https://www.rivm.nl/sites/default/files/2020-12/Whole%20thesis-Jenny%20Deng-23-11-2020.pdf<br>https://www.rivm.nl/bibliotheek/rapporten/2016-0195.pdf<br>https://edepot.wur.nl/550216<br>https://www.who.int/health-topics/food-safety/#tab=tab_1<br>https://edepot.wur.nl/541800<br>https://edepot.wur.nl/199857<br>https://www.rivm.nl/bibliotheek/rapporten/250911006.pdf<br>https://edepot.wur.nl/121239<br>https://edepot.wur.nl/504736<br>https://www.ansnet.org/en/docs/ANEC8BookofAbstracts.pdf#page=148<br>https://edepot.wur.nl/407189food-safety-statement.pdf		
**COUNTRY**	SPAIN		
**Institutional Name**	Spanish Agency for Food Safety and Nutrition<br>Ministry of Agriculture, Fisheries and Food.<br>Ministry of Health	**Web Site included in the Search string**	site:aesan.gob.es<br>site:mapa.gob.es<br>site:sanidad.gob.es
**Documents**	https://www.aesan.gob.es/AECOSAN/docs/documentos/publicaciones/revistas_comite_cientifico/comite_cientifico_19.pdf		
**COUNTRY**	SWITZERLAND		
**Institutional Name**	Federal Food Safety and Veterinary Office<br>Federal Unit for the Food Chain.<br>FOAG.	**Web Site included in the Search string**	site:blv.admin.ch<br>site:blk.admin.ch<br>site:blw.admin.ch
**Documents**	The cost of food consumption across socioeconomic groups in Switzerland estimation of the price of a healthier diet and its impact on nutritional inequality.pdf		

COUNTRY	UNITED KINGDOM		
**Institutional Name**	Government services.<br>Food Standards Scotland.<br>Food Safety Promotion	**Web Site included in the Search string**	site:gov.uk<br>site:foodstandards.gov.scot<br>site:safefood.net
**Documents**	https://www.food.gov.uk/sites/default/files/media/document/Food%20and%20You%202_Wave%206%20Key%20Findings_Final.pdf<br>https://www.food.gov.uk/research/research-projects/kitchen-life-2-literature-review<br>https://assets.publishing.service.gov.uk/media/5d5fc29540f0b607be69a234/20181024-20_04_05-_JSP_456_DCM_Pt_2_Vol_3_-_Ch_3_Food_Safety_Practices_Amnd_013.pdf<br>https://www.food.gov.uk/research/behaviour-and-perception/food-and-you-2-wave-6-key-findings?print=1<br>https://www.food.gov.uk/research/behaviour-and-perception/food-and-you-2-wave-6-key-findings?print=1<br>https://www.food.gov.uk/research/behaviour-and-perception/the-covid-19-consumer-research<br>https://www.food.gov.uk/sites/default/files/media/document/fitnesstoworkguide.pdf<br>https://www.food.gov.uk/sites/default/files/media/document/fs101098fbdinthehometechnicalrep.pdf<br>https://www.food.gov.uk/sites/default/files/media/document/fs241052-report.pdf<br>https://www.foodstandards.gov.scot/downloads/Food_Handlers_-_Fitness_to_work.pdf<br>https://www.foodstandards.gov.scot/downloads/SG018_FSS_Consumer_Tracker_Wave_7_Report.pdf<br>https://www.foodstandards.gov.scot/downloads/Wave_9_Consumer_Tracker.pdf<br>https://www.foodstandards.gov.scot/downloads/Food_handlers_-_Fitness_to_work_v6_-_Final_-_December_2019.pdf<br>https://www.safefood.net/getmedia/a8fff4f5-83c2-4cf3-9ab3-4280fbd5bad1/Volume-1-Final.aspx?ext=.pdf<br>https://www.safefood.net/research-portfolio/search<br>https://www.safefood.net/food-safety/cross-contamination		

**Search String**	ISQ		
**COUNTRY**	ITALY		
**Institutional Name**	Ministry of Health.<br>CREA.<br>Istituto Superiore di Sanità	**Web Site included in the Search string**	site:salute.gov.it<br>site:crea.gov.it<br>site:iss.it
**Documents**	https://www.crea.gov.it/documents/71515/0/Annuario_2016_WEB_DEF.pdf/8eb0e263-2fde-5c0e-c685-dae8fd189fd7?t=1551357444180<br>https://www.crea.gov.it/documents/59764/0/LINEE-GUIDA+DEFINITIVO.pdf/28670db4-154c-0ecc-d187-1ee9db3b1c65?t=1576850671654<br>https://www.crea.gov.it/documents/59764/0/Osservatorio+CREA+O-ERSA+WEB.pdf/c93f0266-e5f7-e2f3-e581-32797328bb3b?t=1559138880594<br>https://www.epicentro.iss.it/tossinfezioni/pdf/MODULISTICA%202021.pdf<br>https://www.epicentro.iss.it/tossinfezioni/pdf/LINEE%20GUIDA%20PER%20LA%20CORRETTA%20GESTIONE%20DELLE%20MALATTIE%20VEICOLATE%20DA%20ALIMENTI.pdf<br>https://www.epicentro.iss.it/tossinfezioni/pdf/lineeguidatoscanatossinfezioni.pdfhttps://www.iss.it/documents/20126/955767/0303.1109071669.pdf/818942a7-640a-e492-5d9a-fb36cc43a370?version=1.1&t=1575578366770&download=true https://www.iss.it/documents/20126/45616/Rapp.+Istisan+00-19.1180605401.pdf/49f2971f-8484-a6fb-4548-c9b7117f514b?t=1581103162160 https://www.epicentro.iss.it/ebp/pdf/report%20alimenti%20apicius4.pdf<br>https://www.iss.it/documents/20126/45616/25.1231840759.pdf/e8181b48-b4ef-3878-c537-11da68f8c58d?t=1581095965479		

**Search String**	SSQ		
**COUNTRY**	SPAIN		
**Institutional Name**	AESAN<br>Department of Agriculture, Fisheries, and Food.<br>Department of Health	**Web Site included in the Search string**	site:aesan.gob.es<br>site:mapa.gob.es<br>site:sanidad.gob.es
**Documents**	https://www.aesan.gob.es/AECOSAN/docs/documentos/publicaciones/revistas_comite_cientifico/comite_cientifico_19.pdf<br>https://www.aesan.gob.es/AECOSAN/docs/documentos/publicaciones/seguridad_alimentaria/seguridad_edu_secundaria.pdf<br>https://www.aesan.gob.es/AECOSAN/web/seguridad_alimentaria/campanyas/campania_verano.htm		

**Search String**	ESQ		
**COUNTRY**	EUROPE		
**Institutional Name**	EUROBAROMETER	**Web Site included in the Search string**	https://europa.eu/eurobarometer/surveys/browse/all
**Documents**	https://europa.eu/eurobarometer/surveys/detail/1476<br>https://europa.eu/eurobarometer/surveys/detail/1054<br>https://europa.eu/eurobarometer/surveys/detail/2241<br>https://www.efsa.europa.eu/it/corporate/pub/eurobarometer22		

**EUROPEAN COUNTRIES analysed with NO RESULTS**<br>**TOT N° Countries: 17**<br>**TOT N° Documents: 0**			
**Search String**	ESQ		
**COUNTRY**	AUSTRIA		
**Institutional Name**	Federal Office for Food Safety<br>Federal Ministry of Social Affairs, health, Care and consumer protection<br>Austrian Agency for Health and Food Safety	**Web Site included in the Search string**	site:baes.gv.at<br>site:sozialministerium.at<br>site:ages.at
**COUNTRY**	BELGIUM		
**Institutional Name**	Health Research Institute<br>Federal Public Service for Health, Food Chain Safety and Environment<br>Federal Agency for the Safety of the Food Chain	**Web Site included in the Search string**	site:sciensano.be<br>site:health.belgium.be<br>site:fasfc.be
**COUNTRY**	CROATIA		
**Institutional Name**	Agency for Agriculture and Food<br>Ministry of Health<br>HZJZ Ministry of Health	**Web Site included in the Search string**	site:hah.hr<br>site:zdravlje.gov.hr<br>site:hzjz.hr

**COUNTRY**	CYPRUS		
**Institutional Name**	Ministry of Health<br>SGL State General Laboratory<br>Public Health Services of the Department of Medical and Public Health Services	**Web Site included in the Search string**	site:moh.gov.cy<br>site:moh.gov.cy/sgl<br>site:moh.gov.cy/Moh/mphs/phs.nsf
**COUNTRY**	CZECH REPUBLIC		
**Institutional Name**	Agriculture and Food Inspection Authority<br>Ministry of Health<br>National Institute of Public Health	**Web Site included in the Search string**	site:szpi.gov.cz<br>site:mzcr.cz<br>site:szu.cz
**COUNTRY**	DENMARK		
**Institutional Name**	Agriculture and Food Inspection Authority.<br>Ministry of Health.<br>National Institute of Public Health.	**Web Site included in the Search string**	site:eng.lbst.dk<br>site:food.dtu.dk<br>site:en.fvm.dk/
**COUNTRY**	ESTONIA		
**Institutional Name**	Agriculture and Food Board.<br>Ministry of Rural Affairs.<br>Veterinary and Food Laboratory	**Web Site included in the Search string**	site:pta.agri.ee<br>site:agri.ee<br>site:labris.agri.ee
**COUNTRY**	NORWAY		
**Institutional Name**	Norwegian Scientific Committee for Food and Environment.<br>NFSA.<br>Government.no	**Web Site included in the Search string**	site:vkm.no<br>site:mattilsynet.no<br>site:Regjeringen.no
**COUNTRY**	POLAND		
**Institutional Name**	Ministry of Agriculture and Rural Development Republic of Poland.<br>National Institute of Public Health.<br>Office of Competition and Consumer Protection	**Web Site included in the Search string**	site:gov.pl/web/agriculture<br>site:pzh.gov.pl<br>site:uokik.gov.pl
**COUNTRY**	PORTUGAL		
**Institutional Name**	General Directorate of Food and Veterinary.<br>Food and Economic Security Authority .<br>Government of Portugal	**Web Site included in the Search string**	site:dgav.pt<br>site:asae.gov.pt<br>site:portugal.gov.pt
**COUNTRY**	ITALY		
**Institutional Name**	Ministry of Health.<br>CREA.<br>Istituto Superiore di Sanità<br>Ministry of Agriculture and Food Sovereignty	**Web Site included in the Search string**	site:salute.gov.it<br>site:crea.gov.it<br>site:iss.it<br>site:politicheagricole.it

**COUNTRY**	ROMANIA		
**Institutional Name**	Ministry of Health.<br>Ministry of Agriculture and Rural Development.<br>The national veterinary health and food safety authority	**Web Site included in the Search string**	site:ms.ro<br>site:madr.ro<br>site:ansvsa.ro
**COUNTRY**	SLOVAKIA		
**Institutional Name**	Ministry of Health.<br>National Agricultural and Food center.<br>Public Health Offices	**Web Site included in the Search string**	site:mzsr.sk<br>site:vup.sk<br>site:uvzsr.sk
**COUNTRY**	SLOVENIA		
**Institutional Name**	Health Inspectorate.<br>Ministry of Health.<br>National Institute of Public Health.	**Web Site included in the Search string**	site:gov.si/drzavni-organi/organi-v-sestavi/zdravstveni-inspektorat<br>site:gov.si<br>site:nijz.si
**COUNTRY**	SWEDEN		
**Institutional Name**	Government.<br>Veterinary Agency.<br>Food Agency	**Web Site included in the Search string**	site:government.se<br>site:sva.se<br>site:livsmedelsverket.se
**COUNTRY**	LATVIA		
**Institutional Name**	BIOR.<br>Disease Prevention and Control Center .<br>Ministry of Health	**Web Site included in the Search string**	site:vm.gov.lv/lv<br>site:bior.lv<br>site:spkc.gov.lv/lv
**COUNTRY**	LITHUANIA		
**Institutional Name**	Ministry of Health.<br>Health Promotion Center.<br>National Food and Veterinary Risk Assessment Institute.<br>State Food and Veterinary Service	**Web Site included in the Search string**	site:sam.lrv.lt<br>site:smlpc.lt<br>site:nmvrvi.lt<br>site:vmvt.lt

INTER-GOVERNMENTAL organizations analysed, with RESULTS<br>TOT N° organizations: 2<br>TOT N° Documents: 17			
**Search String**	ESQ		
**COUNTRY**	intergovernmental organizations operating at the international level		
**Institutional Name**	FAO<br>WHO	**Web Site included in the Search string**	site:fao.org filetype:pdf<br>site:who.int filetype:pdf
**Documents**	https://www.fao.org/fileadmin/templates/food_composition/documents/ABSTRACT_BOOK.pdf<br>https://www.fao.org/3/i5896e/i5896e.pdf<br>https://www.fao.org/fileadmin/templates/ess/documents/icas2/ICAS2.pdf<br>https://www.fao.org/3/cb8667en/cb8667en.pdf<br>https://www.fao.org/3/j6122e/j6122e.pdf<br>https://www.fao.org/fao-who-codexalimentarius/sh-proxy/en/?lnk=1&url=https%253A%252F%252Fworkspace.fao.org%252Fsites%252Fcodex%252FStandards%252FCXC%2B80-2020%252FCXC_080e.pdf<br>https://www.fao.org/3/y4392e/y4392e.pdf<br>https://www.fao.org/3/i7321en/I7321EN.pdf<br>https://www.fao.org/3/i8013e/i8013e.pdf<br>https://www.fao.org/3/i4690e/i4690e.pdf<br>https://www.fao.org/3/i8240en/I8240EN.pdf<br>https://www.who.int/docs/default-source/food-safety/jemra/call-for-consultation/methodology-report-public-comments.pdf<br>https://www.afro.who.int/sites/default/files/2017-06/fao_who_conf_national_food_safety_africa.pdf<br>https://extranet.who.int/nutrition/gina/sites/default/filesstore/SLE%202020%20NCD%20strategic%20plan%202020-2024.pdf<br>9291562293-eng (1).pdf<br>9789241547369_eng.pdf		

NOT EUROPEAN COUNTRIES analysed, with RESULTS<br>TOT N° countries: 4<br>TOT N° Documents: 32			
**Search String**	ESQ		
**COUNTRY**	USA		
**Institutional Name**	FDS<br>CDC<br>USDA FSIS	**Web Site included in the Search string**	site:fda.gov filetype:pdf<br>site:cdc.gov<br>site:fsis.usda.gov
**Documents**	https://www.fda.gov/media/101366/download<br>https://www.fda.gov/media/146532/download<br>https://www.fda.gov/media/89145/download<br>https://www.cdc.gov/mmwr/preview/mmwrhtml/mm5934a2.htm<br>Safe Food Handling and Preparation | Food Safety and Inspection Service (usda.gov)<br>Food Safety Consumer Research Project: Web-based Survey on Food Safety Behaviors and Consumer Education (usda.gov)<br>Cooking with Microwave Ovens | Food Safety and Inspection Service (usda.gov)<br>Food Safety Basics | Food Safety and Inspection Service (usda.gov)<br>Home | Food Safety and Inspection Service (usda.gov)<br>Food Safety Consumer Research Project: Meal Preparation Experiment Related to Thermometer Use May 18, 2018 (usda.gov)<br>Keep Food Safe! Food Safety Basics | Food Safety and Inspection Service (usda.gov)<br>Observational Study Year 3 Web Survey (usda.gov)<br>Food Product Dating | Food Safety and Inspection Service (usda.gov)<br>How Temperatures Affect Food | Food Safety and Inspection Service (usda.gov)		
**COUNTRY**	CANADA		
**Institutional Name**	Health Canada<br>PHA<br>CFIA	**Web Site included in the Search string**	site:canada.ca<br>phac-aspc.gc.ca<br>inspection.canada.ca
**Documents**	https://www.canada.ca/en/public-health/services/infectious-diseases/centre-food-borne-environmental-zoonotic-infectious-diseases/national-single-day-food-consumption-report.html<br>https://inspection.canada.ca/en/food-safety-industry/toolkit-food-businesses/sfcr-handbook-food-businesses<br>https://www.canada.ca/content/dam/esdc-edsc/documents/programs/ei/ei-list/reports/monitoring2020/2019-2020_EI_MAR-EN.pdf<br>National Single Day Food Consumption Report - Canada.ca<br>Understanding the Safe Food for Canadians Regulations: A handbook for food businesses - inspection.canada.ca		

**COUNTRY**	AUSTRIALIA AND NEW ZEALAND		
**Institutional Name**	Food Standards	**Web Site included in the Search string**	foodstandards.gov.au
**Documents**	https://www.foodstandards.gov.au/foodsafety/standards/Pages/Food-Safety-Standards-(Chapter-3).aspx<br>https://www.foodstandards.gov.au/publications/documents/Appendix_E_%20Consumer_Attitudes_Survey-3.pdf<br>https://www.foodstandards.gov.au/consumer/safety/faqsafety/pages/foodsafetyfactsheets/foodsafetypracticesa70.aspx P282_Poultry _ DAR Attach3.doc<br>https://www.foodstandards.gov.au/industry/safetystandards/documents/Guide%20321%20FoodSafetyPrograms-WEB.pdf<br>https://www.foodstandards.gov.au/code/proposals/Documents/SD2%20FINAL_2nd%20CFS%20Micro%20RA%20P1052%20with%20appendices_ref%20unlinked.pdf<br>https://www.foodstandards.gov.au/code/proposals/documents/p1007%20ppps%20for%20raw%20milk%201ar%20sd1%20cow%20milk%20risk%20assessment.pdf<br>https://www.foodstandards.gov.au/code/proposals/documents/P1007%20PPPS%20for%20raw%20milk%201AR%20SD3%20Cheese%20Risk%20Assessment.pdf<br>https://www.foodstandards.gov.au/publications/documents/2007%20NFHS%20Interpretive%20Summary%20FINAL.pdf<br>https://www.foodstandards.gov.au/publications/documents/Consumer%20Attitudes%20Survey.pdf<br>https://www.foodstandards.gov.au/publications/Documents/Safe%20Food%20Australia/FSANZ%20Safe%20Food%20Australia_WEB.pdf<br>https://www.foodstandards.gov.au/publications/documents/complete_safefood.pdf<br>https://www.foodstandards.gov.au/code/proposals/documents/P1007%20PPPS%20for%20raw%20milk%201AR%20SD2%20Goat%20milk%20Risk%20Assessment.pdf<br>https://www.foodstandards.gov.au/code/proposals/documents/DAR_P296_Dairy_PPPS_Attach_2%20_App%20_1_6.pdf<br>https://www.foodstandards.gov.au/industry/safetystandards/safetypractices/skills/Pages/default.aspx		
**Search String**	SSQ		
**COUNTRY**	CHILE		
**Institutional Name**	ACHIPIA	**Web Site included in the Search string**	site:achipia.gob.cl
**Documents**	https://www.achipia.gob.cl/wp-content/uploads/2021/10/MANUAL_MANIPULADORES_FORMADORES.pdf		

NOT EUROPEAN COUNTRIES analysed, with NO RESULTS<br>TOT N° countries: 11<br>TOT N° Documents: 0			
**Search String**	ESQ		
**COUNTRY**	SAUDI ARABIA		
**Institutional Name**	SFDS	**Web Site included in the Search string**	site:sfda.gov.sa
**COUNTRY**	SOUTH KOREA		
**Institutional Name**	MFDF	**Web Site included in the Search string**	site:mfds.go.kr
**COUNTRY**	JAPAN		
**Institutional Name**	Ministry of health<br>MAFF	**Web Site included in the Search string**	site:mhlw.go.jp<br>site:maff.go.jp
**COUNTRY**	PANAMA		
**Institutional Name**	APA	**Web Site included in the Search string**	site:fmm.com.pa
**COUNTRY**	GUYANA		
**Institutional Name**	Ministry of Agriculture.<br>Ministry of Health	**Web Site included in the Search string**	site:agriculture.gov.gy<br>site:health.gov.gy
**COUNTRY**	URUGUAY		
**Institutional Name**	Food safety governmental bodies	**Web Site included in the Search string**	NOT FOUND
**COUNTRY**	OMAN		
**Institutional Name**	Ministry of agriculture, Fisheries and Water Resources	**Web Site included in the Search string**	site:omanportal.gov.om
**COUNTRY**	UNITED ARAB EMIRATES		
**Institutional Name**	Official portal of the UAE Government	**Web Site included in the Search string**	site:u.ae/en
**COUNTRY**	QATAR		
**Institutional Name**	Food safety department	**Web Site included in the Search string**	site:emsfsa.moph.gov.qa
**COUNTRY**	KUWAIT		
**Institutional Name**	Food safety governmental bodies	**Web Site included in the Search string**	NOT FOUND
**COUNTRY**	ISRAEL		
**Institutional Name**	National Food Safety	**Web Site included in the Search string**	site:gov.il/en/

Supplementary Materials S4 **Data extraction Form**

	A	B	C	D	E	F	G	H	I	J
1	**Study ID**	**Title**	**Reviewer Name**	**Reference**	**1. Article type**	**2. Data collection methods**	**3. Testing**	**4. Data analysis method**	**5. Target population**	**6. Survey question type**
2										
3										
4										

K	L	M	N	O	P	Q	R	S
**7. Cash/voucher compensation**	**8. Sample size**	**9. Survey aims**	**10. Survey Conclusions /Discussion**	**11. Survey results analysis**	**12. Survey administration /distribution**	**13. Geographical location**	**14. Food safety topics**	**15. Start/end date**

Table S1

Supplementary Table S1. Detailed distribution of food safety topic categories across scientific and grey literature. The table reports the number of studies addressing individual food safety topics (single-topic categories) and specific combinations of topics within mixed-topic categories (studies addressing two, three, or four topic areas). Topic areas include microbiological, chemical, nutritional, home food safety practices (HFSP), and lifestyle-related aspects.

Topics	Scientific	Grey
**Single topic (total number)**	**112**	**11**
Microbiological	81	4
Nutritional	9	5
Other	13	1
Chemical	2	1
Home Food Safety Practices - HFSP	7	0
**Mixed: 2 topics (total number)**	**91**	**13**
Microbiological+HFSP	48	4
Nutritional+Lifestyle	32	1
Nutritional+HFSP	3	0
Chemical+Nutritional	2	0
Chemical+HFSP	2	0
Microbiological+Nutritional	2	4
Microbiological+Other	1	0
Nutritional+Other	1	1
HFSP+Other	0	3
**Mixed: 3 topics (total number)**	**41**	**3**
Microbiological+Nutritional+Lifestyle	33	0
Chemical+Nutritional+Lifestyle	2	0
Microbiological+ Lifestyle+Other	2	2
Nutritional+Lifestyle+HFSP	2	0
Nutritional+Lifestyle+Other	2	0
Microbiological+Nutritional+Other	0	1
**Mixed: 4 topics (total number)**	**4**	**0**
Microbiological+Chemical+Nutritional+Lifestyle	3	0
Microbiological+Chemical+Nutritional+Other	1	0

Supplementary Materials S5 **Citation list of 274 relevant articles captured in this Scoping review**

5.1 Scientific literature (247 papers)

1. Anderson, J.B.; Shuster, T.A.; Hansen, K.E.; Levy, A.S.; Volk, A. A Camera's View of Consumer Food-Handling Behaviors. J Am Diet Assoc. **2004**, *104*, 186–191, doi:10.1016/j.jada.2003.11.010.
2. Cho, T.J.; Kim, S.A.; Kim, H.W.; Rhee, M.S. A Closer Look at Changes in High-Risk Food-Handling Behaviors and Perceptions of Primary Food Handlers at Home in South Korea across Time. *Foods*

2020, *9*, 1457, doi:10.3390/foods9101457.
3. Wong, L.Y.; Francis, S.L.; Genschel, U.; Arthur, A.; Xu, F.; Weidauer, L.; Monroe-Lord, L.; Ventura-Marra, M.; Sahyoun, N.R.; Kendall, C. A Cross-Sectional Assessment of Food Practices, Physical Activity Levels, and Stress Levels in Middle Age and Older Adults' during the COVID-19 Pandemic. *J Public Health (Berl.)* **2023**, *31*, 1567–1583, doi:10.1007/s10389-022-01742-y.
4. Osaili, T.; Shaker Obaid, R.; Taha, S.; Kayyaal, S.; Ali, R.; Osama, M.; Alajmi, R.; Al-Nabulsi, A.A.; Olaimat, A.; Hasan, F.; et al. A Cross-Sectional Study on Food Safety Knowledge amongst Domestic Workers in the UAE. *Br. Food J.* **2022**, *124*, 1009–1021, doi:10.1108/BFJ-12-2020-1124.
5. Li, Z.; Liu, H.; Tu, W. A Generalized Semiparametric Mixed Model for Analysis of Multivariate Health Care Utilization Data. *Stat Methods Med Res* **2017**, *26*, 2909–2918, doi:10.1177/0962280215615159.
6. Stenger, K.M.; Ritter-Gooder, P.K.; Perry, C.; Albrecht, J.A. A Mixed Methods Study of Food Safety Knowledge, Practices and Beliefs in Hispanic Families with Young Children. *Appetite* **2014**, *83*, 194–201, doi:10.1016/j.appet.2014.08.034.
7. Herbold, N.H.; Scott, E. A Pilot Study Describing Infant Formula Preparation and Feeding Practices. *Int. J. Environ. Health Res.***2008**, *18*, 451–459, doi:10.1080/09603120802272185.
8. Blake, C.E.; Fisher, J.O.; Ganter, C.; Younginer, N.; Orloski, A.; Blaine, R.E.; Bruton, Y.; Davison, K.K. A Qualitative Study of Parents' Perceptions and Use of Portion Size Strategies for Preschool Children's Snacks. *Appetite* **2015**, *88*, 17–23, doi:10.1016/j.appet.2014.11.005.
9. Millman, C.; Rigby, D.; Jones, D.; Edwards-Jones, G. A Real-Time Test of Food Hazard Awareness. *Br. Food J.* **2015**, *117*, 2112–2128, doi:10.1108/BFJ-09-2014-0317.
10. Patrick, H.; Nicklas, T.A. A Review of Family and Social Determinants of Children's Eating Patterns and Diet Quality. *J Am Coll Nutr.***2005**, *24*, 83–92, doi:10.1080/07315724.2005.10719448.
11. Yang, L.; Arnold, N.L.; Drape, T.; Williams, R.C.; Archibald, T.; Chapman, B.; Boyer, R. A Survey of United States Consumer Awareness, Purchasing, and Handling of Mechanically Tenderized Beef Products. *Food Control* **2021**, *120*, 107505, doi:10.1016/j.foodcont.2020.107505.
12. Kaiser, M.L.; Carr, J.K.; Fontanella, S. A Tale of Two Food Environments: Differences in Food Availability and Food Shopping Behaviors between Food Insecure and Food Secure Households. *J. Hunger Environ. Nutr.***2019**, *14*, 297–317, doi:10.1080/19320248.2017.1407723.
13. Langsrud, S.; Veflen, N.; Allison, R.; Crawford, B.; Izsó, T.; Kasza, G.; Lecky, D.; Nicolau, A.I.; Scholderer, J.; Skuland, S.E.; et al. A Trans Disciplinary and Multi Actor Approach to Develop High Impact Food Safety Messages to Consumers: Time for a Revision of the WHO - Five Keys to Safer Food? *Trends Food Sci. Technol.* **2023**, *133*, 87–98, doi:10.1016/j.tifs.2023.01.018.
14. Kowalska, M.; Żbikowska, A.; Onacik-Gür, S.; Kowalska, D. Acrylamide in Food Products – Eating Habits and Consumer Awareness among Medical School Students. *Ann Agric Environ Med.* **2017**, *24*, 570–574, doi:10.5604/12321966.1232764.
15. SteelFisher, G.; Blendon, R.; Hero, J.; Ben-Porath, E. Adoption of Self-Protective Behaviors in Response to a Foodborne Illness Outbreak: Perspectives of Older Adults. *J. Food Saf.***2013**, *33*, 149–162, doi:10.1111/jfs.12035.
16. De Backer, C.; Teunissen, L.; Cuykx, I.; Decorte, P.; Pabian, S.; Gerritsen, S.; Matthys, C.; Al Sabbah, H.; Van Royen, K.; the Corona Cooking Survey Study Group An Evaluation of the COVID-19 Pandemic and Perceived Social Distancing Policies in Relation to Planning, Selecting, and Preparing Healthy Meals: An Observational Study in 38 Countries Worldwide. *Front. Nutr.* **2021**, *7*, 621726, doi:10.3389/fnut.2020.621726.
17. Masson, M.; Delarue, J.; Blumenthal, D. An Observational Study of Refrigerator Food Storage by Consumers in Controlled Conditions. *Food Qual. Prefer.* **2017**, *56*, 294–300, doi:10.1016/j.foodqual.2016.06.010.
18. Evans, E.W.; Redmond, E.C. Analysis of Older Adults' Domestic Kitchen Storage Practices in the United Kingdom: Identification of Risk Factors Associated with Listeriosis. *J. Food Prot.* **2015**, *78*, 738–745, doi:10.4315/0362-028X.JFP-14-527.
19. Mari, S.; Tiozzo, B.; Capozza, D.; Ravarotto, L. Are You Cooking Your Meat Enough? The Efficacy

of the Theory of Planned Behavior in Predicting a Best Practice to Prevent Salmonellosis. *Food Res. Int.* **2012**, *45*, 1175–1183, doi:10.1016/j.foodres.2011.06.028.
20. Banna, J.C.; Townsend, M.S. Assessing Factorial and Convergent Validity and Reliability of a Food Behaviour Checklist for Spanish-Speaking Participants in US Department of Agriculture Nutrition Education Programmes. *Public Health Nutr.* **2011**, *14*, 1165–1176, doi:10.1017/S1368980010003058.
21. Redmond, E.C.; Griffith, C.J. Assessment of Consumer Food Safety Education Provided by Local Authorities in the UK. *Br. Food J.* **2006**, *108*, 732–752, doi:10.1108/00070700610688377.
22. Nitu, I.; Rus, V.A.; Sipos, R.S.; Nyulas, T.; Cherhat, M.P.; Ruta, F.; Tita, C.C.N. Assessment of Eating Behavior During the COVID-19 Pandemic Period. A Pilot Study. *J. Interdiscip. Med.* **2021**, *6*, 67–73, doi:10.2478/jim-2021-0024.
23. Hulin, M.; Bemrah, N.; Nougadère, A.; Volatier, J.L.; Sirot, V.; Leblanc, J.C. Assessment of Infant Exposure to Food Chemicals: The French Total Diet Study Design. *Food Addit. Contam.: Part A* **2014**, 1–14, doi:10.1080/19440049.2014.921937.
24. Alkerwi, A.; Sauvageot, N.; Malan, L.; Shivappa, N.; Hébert, J. Association between Nutritional Awareness and Diet Quality: Evidence from the Observation of Cardiovascular Risk Factors in Luxembourg (ORISCAV-LUX) Study. *Nutrients* **2015**, *7*, 2823–2838, doi:10.3390/nu7042823.
25. Talas, C.; Uçar, A.; Özfer Özçelik, A. Attitudes of Women towards Food Safety. *Br. Food J.* **2010**, *112*, 1115–1123, doi:10.1108/00070701011080249.
26. Mateus, T.; Maia, R.L.; Teixeira, P. Awareness of Listeriosis among Portuguese Pregnant Women. *Food Control* **2014**, *46*, 513–519, doi:10.1016/j.foodcont.2014.06.012.
27. Nesbitt, A.; Thomas, M.K.; Marshall, B.; Snedeker, K.; Meleta, K.; Watson, B.; Bienefeld, M. Baseline for Consumer Food Safety Knowledge and Behaviour in Canada. *Food Control* **2014**, *38*, 157–173, doi:10.1016/j.foodcont.2013.10.010.
28. Evans, E.W.; Redmond, E.C. Behavioral Risk Factors Associated with Listeriosis in the Home: A Review of Consumer Food Safety Studies. *J. Food Prot.* **2014**, *77*, 510–521, doi:10.4315/0362-028X.JFP-13-238.
29. Atoloye, A.T.; Durward, C. Being Motivated by Nutrition Is Associated With Healthy Home Food Environment of Supplemental Nutrition Assistance Program (SNAP) Recipients. *J. Nutr. Educ. Behav.***2019**, *51*, 101–106, doi:10.1016/j.jneb.2018.07.013.
30. Danyliw, A.D.; Vatanparast, H.; Nikpartow, N.; Whiting, S.J. Beverage Intake Patterns of Canadian Children and Adolescents. *Public Health Nutr.* **2011**, *14*, 1961–1969, doi:10.1017/S1368980011001091.
31. Tatlow-Golden, M.; Hennessy, E.; Dean, M.; Hollywood, L. 'Big, Strong and Healthy'. Young Children's Identification of Food and Drink That Contribute to Healthy Growth. *Appetite* **2013**, *71*, 163–170, doi:10.1016/j.appet.2013.08.007.
32. Muringai, V.; Goddard, E. Bovine Spongiform Encephalopathy, Risk Perceptions, and Beef Consumption: Differences Between Canada and Japan. *J. Toxicol. Environ. Health, Part A* **2011**, *74*, 167–190, doi:10.1080/15287394.2011.529327.
33. Phang, H.S.; Bruhn, C.M. Burger Preparation: What Consumers Say and Do in the Home. *J. Food Prot.* **2011**, *74*, 1708–1716, doi:10.4315/0362-028X.JFP-10-417.
34. Myintzaw, P.; Moran, F.; Jaiswal, A.K. Campylobacteriosis, Consumer's Risk Perception, and Knowledge Associated with Domestic Poultry Handling in Ireland. *J. Food Saf.* **2020**, *40*, e12799, doi:10.1111/jfs.12799.
35. Marklinder, I.; Magnusson, M.; Nydahl, M. CHANCE: A Healthy Lifestyle in Terms of Food Handling and Hygiene. *Br. Food J.* **2013**, *115*, 223–234, doi:10.1108/00070701311302203.
36. Cho, T.J.; Kim, S.A.; Kim, H.W.; Park, S.M.; Rhee, M.S. Changes in Consumers' Food Purchase and Transport Behaviors over a Decade (2010 to 2019) Following Health and Convenience Food Trends. *IJERPH* **2020**, *17*, 5448, doi:10.3390/ijerph17155448.
37. Berta Vanrullen, I.; Volatier, J.-L.; Bertaut, A.; Dufour, A.; Dallongeville, J. Characteristics of Energy Intake Under-Reporting in French Adults. *Br. Food J.* **2014**, *111*, 1292–1302, doi:10.1017/S0007114513003759.
38. Torres, A.; Langenhoven, P.; Behe, B.K. Characterizing the U.S. Melon Market. *horts* **2020**, *55*, 795–

803, doi:10.21273/HORTSCI14859-20.
39. Almanza, B.A.; Namkung, Y.; Ismail, J.A.; Nelson, D.C. Clients' Safe Food-Handling Knowledge and Risk Behavior in a Home-Delivered Meal Program. *J Am Diet Assoc* **2007**, *107*, 816–821, doi:10.1016/j.jada.2007.02.043.
40. Zamanillo Campos, R.; Rovira Boixadera, L.; Rendo Urteaga, T. Common Practices and Beliefs in the Preparation of Complementary Infant Feeding in a Spanish Sample: A Cross-Sectional Study. *Nutr Hosp* **2021**, doi:10.20960/nh.03527.
41. Abbot, J.M.; Byrd-Bredbenner, C.; Schaffner, D.; Bruhn, C.M.; Blalock, L. Comparison of Food Safety Cognitions and Self-Reported Food-Handling Behaviors with Observed Food Safety Behaviors of Young Adults. *Eur J Clin Nutr* **2009**, *63*, 572–579, doi:10.1038/sj.ejcn.1602961.
42. Torres, J.; Voisier, A.; Berríos, I.; Pitto, N.; Agüero, S.D. Conocimiento y Aplicación En Prácticas Higiénicas En La Elaboración de Alimentos y Auto-Reporte de Intoxicaciones Alimentarias En Hogares Chilenos. *Rev. chil. infectol.* **2018**, *35*, 483–489, doi:10.4067/s0716-10182018000500483.
43. Frenzen, P.D.; Debess, E.E.; Hechemy, K.E.; Kassenborg, H.; Kennedy, M.; Mccombs, K.; Mcnees, A. Consumer Acceptance of Irradiated Meat and Poultry in the United States. *J. Food Prot.* **2001**, *64*, 2020–2026, doi:10.4315/0362-028X-64.12.2020.
44. Redmond, E.C.; Griffith, C.J. CONSUMER ATTITUDES AND PERCEPTIONS TOWARDS MICROBIAL FOOD SAFETY IN THE DOMESTIC KITCHEN. *J. Food Saf.* **2004**, *24*, 169–194, doi:10.1111/j.1745-4565.2004.tb00383.x.
45. Badrie, N.; Gobin, A.; Dookeran, S.; Duncan, R. Consumer Awareness and Perception to Food Safety Hazards in Trinidad, West Indies. *Food Control* **2006**, *17*, 370–377, doi:10.1016/j.foodcont.2005.01.003.
46. Redmond, E.C.; Griffith, C.J. Consumer Food Handling in the Home: A Review of Food Safety Studies. *J. Food Prot.* **2003**, *66*, 130–161, doi:10.4315/0362-028X-66.1.130.
47. Bergsma, N.J.; Fischer, A.R.H.; Van Asselt, E.D.; Zwietering, M.H.; De Jong, A.E.I. Consumer Food Preparation and Its Implication for Survival of *Campylobacter Jejuni* on Chicken. *Br. Food J.* **2007**, *109*, 548–561, doi:10.1108/00070700710761536.
48. Patil, S.R.; Cates, S.; Morales, R. Consumer Food Safety Knowledge, Practices, and Demographic Differences: Findings from a Meta-Analysis. *J. Food Prot.* **2005**, *68*, 1884–1894, doi:10.4315/0362-028X-68.9.1884.
49. Kennedy, J.; Jackson, V.; Cowan, C.; Blair, I.; McDowell, D.; Bolton, D. Consumer Food Safety Knowledge: Segmentation of Irish Home Food Preparers Based on Food Safety Knowledge and Practice. *Br. Food J.* **2005**, *107*, 441–452, doi:10.1108/00070700510606864.
50. Gkana, E.N.; Nychas, G.E. Consumer Food Safety Perceptions and Self-reported Practices in Greece. *Int J Consumer Studies* **2018**, *42*, 27–34, doi:10.1111/ijcs.12391.
51. Kosa, K.M.; Cates, S.C.; Karns, S.; Godwin, S.L.; Chambers, D. Consumer Home Refrigeration Practices: Results of a Web-Based Survey. *J. Food Prot.* **2007**, *70*, 1640–1649, doi:10.4315/0362-028X-70.7.1640.
52. Lee, J.-K.; Kim, E.-H.; Lee, M.-A. Consumer Hygiene Practices Regarding the Use of Home Refrigerators to Store Meat in the Capital Area of Korea. *Korean J. Food Sci. Anim. Resour.* **2013**, *33*, 149–154, doi:10.5851/kosfa.2013.33.2.149.
53. Verbeke, W.; Sioen, I.; Pieniak, Z.; Van Camp, J.; De Henauw, S. Consumer Perception versus Scientific Evidence about Health Benefits and Safety Risks from Fish Consumption. *Public Health Nutr.* **2005**, *8*, 422–429, doi:10.1079/PHN2004697.
54. Redmond, E.C.; Griffith, C.J. Consumer Perceptions of Food Safety Risk, Control and Responsibility. *Appetite* **2004**, *43*, 309–313, doi:10.1016/j.appet.2004.05.003.
55. Yeargin, T.A.; Lin, Z.; Do Prado, I.; Sirsat, S.A.; Gibson, K.E. Consumer Practices and Perceptions Regarding the Purchasing and Handling of Microgreens in the United States. *Food Control* **2023**, *145*, 109470, doi:10.1016/j.foodcont.2022.109470.
56. Møretrø, T.; Nguyen-The, C.; Didier, P.; Maître, I.; Izsó, T.; Kasza, G.; Skuland, S.E.; Cardoso, M.J.; Ferreira, V.B.; Teixeira, P.; et al. Consumer Practices and Prevalence of Campylobacter, Salmonella

and Norovirus in Kitchens from Six European Countries. *Int. J. Food Microbiol.* **2021**, *347*, 109172, doi:10.1016/j.ijfoodmicro.2021.109172.
57. Kosa, K.M.; Cates, S.C.; Bradley, S.; Godwin, S.; Chambers, D. Consumer Shell Egg Consumption and Handling Practices: Results from a National Survey. *J. Food Prot.* **2015**, *78*, 1312–1319, doi:10.4315/0362-028X.JFP-14-574.
58. Krystallis, A.; Chryssochoidis, G.; Scholderer, J. Consumer-Perceived Quality in 'Traditional' Food Chains: The Case of the Greek Meat Supply Chain. *Appetite* **2007**, *48*, 54–68, doi:10.1016/j.appet.2006.06.003.
59. Kosa, K.M.; Cates, S.C.; Bradley, S.; Edgar Chambers, I.V.; Godwin, S. Consumer-Reported Handling of Raw Poultry Products at Home: Results from a National Survey. *J. Food Prot.* **2015**, *78*, 180–186, doi:10.4315/0362-028X.JFP-14-231.
60. Angelillo, I.F.; Foresta, M.R.; Scozzafava, C.; Pavia, M. Consumers and Foodborne Diseases: Knowledge, Attitudes and Reported Behavior in One Region of Italy. *Int. J. Food Microbiol.* **2001**, *64*, 161–166, doi:10.1016/S0168-1605(00)00451-7.
61. Yu, H.; Lin, Z.; Lin, M.S.; Neal, J.A.; Sirsat, S.A. Consumers' Knowledge and Handling Practices Associated with Fresh-Cut Produce in the United States. *Foods* **2022**, *11*, 2167, doi:10.3390/foods11142167.
62. Odwin, R.; Badrie, N. Consumers' Perceptions and Awareness of Food Safety Practices in Barbados and Trinidad, West Indies – a Pilot Study. *Int J Consumer Studies* **2008**, *32*, 394–398, doi:10.1111/j.1470-6431.2008.00675.x.
63. Boodhu, A.; Badrie, N.; Sookdhan, J. Consumers' Perceptions and Awareness of Safe Food Preparation Practices at Homes in Trinidad, West Indies. *Int J Consumer Studies* **2008**, *32*, 41–48, doi:10.1111/j.1470-6431.2007.00634.x.
64. Tiozzo, B.; Mari, S.; Ruzza, M.; Crovato, S.; Ravarotto, L. Consumers' Perceptions of Food Risks: A Snapshot of the Italian Triveneto Area. *Appetite* **2017**, *111*, 105–115, doi:10.1016/j.appet.2016.12.028.
65. Khouryieh, M.; Khouryieh, H.; Daday, J.K.; Shen, C. Consumers' Perceptions of the Safety of Fresh Produce Sold at Farmers' Markets. *Food Control* **2019**, *105*, 242–247, doi:10.1016/j.foodcont.2019.06.003.
66. Lando, A.M.; Bazaco, M.C.; Chen, Y. Consumers' Use of Personal Electronic Devices in the Kitchen. *J. Food Prot.* **2018**, *81*, 437–443, doi:10.4315/0362-028X.JFP-17-172.
67. Tooby, M.; Morton, V.; Nesbitt, A.; Ciampa, N.; Thomas, M.K. Consumption of High-Risk Foods in the Canadian Population, Foodbook Study, 2014 to 2015. *J. Food Prot.* **2021**, *84*, 1925–1936, doi:10.4315/JFP-21-101.
68. Bremer, V.; Bocter, N.; Rehmet, S.; Klein, G.; Breuer, T.; Ammon, A. Consumption, Knowledge, and Handling of Raw Meat: A Representative Cross-Sectional Survey in Germany, March 2001. *J. Food Prot.* **2005**, *68*, 785–789, doi:10.4315/0362-028X-68.4.785.
69. Langsrud, S.; Sørheim, O.; Skuland, S.E.; Almli, V.L.; Jensen, M.R.; Grøvlen, M.S.; Ueland, Ø.; Møretrø, T. Cooking Chicken at Home: Common or Recommended Approaches to Judge Doneness May Not Assure Sufficient Inactivation of Pathogens. *PLoS ONE* **2020**, *15*, e0230928, doi:10.1371/journal.pone.0230928.
70. Neumark-Sztainer, D.; Wall, M.; Perry, C.; Story, M. Correlates of Fruit and Vegetable Intake among Adolescents. *Preventive Medicine* **2003**, *37*, 198–208, doi:10.1016/S0091-7435(03)00114-2.
71. Kennedy, J.; Nolan, A.; Gibney, S.; O'Brien, S.; McMahon, M.A.S.; McKenzie, K.; Healy, B.; McDowell, D.; Fanning, S.; Wall, P.G. Deteminants of Cross-contamination during Home Food Preparation. . *Br. Food J.* **2011**, *113*, 280–297, doi:10.1108/00070701111105349.
72. Cairnduff, V.; Dean, M.; Koidis, A. Development of the Consumer Refrigerator Safety Questionnaire: A Measure of Consumer Perceptions and Practices. *J. Food Prot.* **2016**, *79*, 1609–1615, doi:10.4315/0362-028X.JFP-15-462.
73. Storz, M.A.; Müller, A.; Lombardo, M. Diet and Consumer Behavior in U.S. Vegetarians: A National Health and Nutrition Examination Survey (NHANES) Data Report. *IJERPH* **2021**, *19*, 67, doi:10.3390/ijerph19010067.

74. Deierlein, A.L.; Litvak, J.; Liu, C.; Stein, C.R. Diet Quality, Diet-Related Factors and Disability Status among Male Adults of Reproductive Age in the USA. *Public Health Nutr.* **2023**, *26*, 1976–1985, doi:10.1017/S1368980023001222.
75. Hartman, T.J.; Haardörfer, R.; Whitaker, L.L.; Addison, A.; Zlotorzynska, M.; Gazmararian, J.A.; Kegler, M.C. Dietary and Behavioral Factors Associated with Diet Quality among Low-Income Overweight and Obese African American Women. *J. Am. Coll. Nutr.* **2015**, *34*, 416–424, doi:10.1080/07315724.2014.982305.
76. Robinson-Oghogho, J.N.; Thorpe, R.J.; Neff, R.A. Dietary Behaviors among New Users of Meal-Kit Services during the Early Months of the COVID-19 Pandemic. *Nutrients* **2022**, *14*, 3953, doi:10.3390/nu14193953.
77. Hoteit, M.; Mortada, H.; Al-Jawaldeh, A.; Mansour, R.; Yazbeck, B.; AlKhalaf, M.; Bookari, K.; Tayyem, R.; Al-Awwad, N.J.; Al Sabbah, H.; et al. Dietary Diversity in the Eastern Mediterranean Region Before and During the COVID-19 Pandemic: Disparities, Challenges, and Mitigation Measures. *Front. Nutr.* **2022**, *9*, 813154, doi:10.3389/fnut.2022.813154.
78. Mitakakis, T.Z.; Wolfe, R.; Sinclair, M.I.; Fairley, C.K.; Leder, K.; Hellard, M.E. Dietary Intake and Domestic Food Preparation and Handling as Risk Factors for Gastroenteritis: A Case-Control Study. *Epidemiol. Infect.* **2004**, *132*, 601–606, doi:10.1017/S0950268804002365.
79. Sharpe, P.A.; Whitaker, K.; Alia, K.A.; Wilcox, S.; Hutto, B. Dietary Intake, Behaviors and Psychosocial Factors among Women from Food-Secure and Food-Insecure Households. *Ethn Dis* **2016**, *26*, 139, doi:10.18865/ed.26.2.139.
80. Ishitsuka, K.; Sasaki, S.; Mezawa, H.; Konishi, M.; Igarashi, M.; Yamamoto-Hanada, K.; Nakayama, S.F.; Ohya, Y. Dietary Supplement Use in Elementary School Children: A Japanese Web-Based Survey. *Environ Health Prev Med* **2021**, *26*, 63, doi:10.1186/s12199-021-00985-7.
81. Parry, S.M.; Miles, S.; Tridente, A.; Palmer, S.R.; South and East Wales Infectious Disease Group Differences in Perception of Risk Between People Who Have and Have Not Experienced *Salmonella* Food Poisoning. *Risk Analysis* **2004**, *24*, 289–299, doi:10.1111/j.0272-4332.2004.00429.x.
82. Møretrø, T.; Moen, B.; Almli, V.L.; Teixeira, P.; Ferreira, V.B.; Åsli, A.W.; Nilsen, C.; Langsrud, S. Dishwashing Sponges and Brushes: Consumer Practices and Bacterial Growth and Survival. *Int. J. Food Microbiol.***2021**, *337*, 108928, doi:10.1016/j.ijfoodmicro.2020.108928.
83. Vanhelst, J.; Béghin, L.; Duhamel, A.; De Henauw, S.; Ruiz, J.R.; Kafatos, A.; Androutsos, O.; Widhalm, K.; Mauro, B.; Sjöström, M.; et al. Do Adolescents Accurately Evaluate Their Diet Quality? The HELENA Study. *Clinical Nutrition* **2017**, *36*, 1669–1673, doi:10.1016/j.clnu.2016.10.019.
84. Evans, M.R.; Sarvotham, T.; Thomas, D.R.; Howard, A.J. Domestic and Travel-Related Foodborne Gastrointestinal Illness in a Population Health Survey. *Epidemiol. Infect.* **2006**, *134*, 686–693, doi:10.1017/S0950268805005790.
85. Kendall, H.; Kuznesof, S.; Seal, C.; Dobson, S.; Brennan, M. Domestic Food Safety and the Older Consumer: A Segmentation Analysis. *Food Qual. Prefer.* **2013**, *28*, 396–406, doi:10.1016/j.foodqual.2012.11.006.
86. Evans, E.W.; Redmond, E.C. Domestic Kitchen Microbiological Contamination and Self-Reported Food Hygiene Practices of Older Adult Consumers. *J. Food Prot.* **2019**, *82*, 1326–1335, doi:10.4315/0362-028X.JFP-18-533.
87. Lagendijk, E.; Asséré, A.; Derens, E.; Carpentier, B. Domestic Refrigeration Practices with Emphasis on Hygiene: Analysis of a Survey and Consumer Recommendations. *J. Food Prot.* **2008**, *71*, 1898–1904, doi:10.4315/0362-028X-71.9.1898.
88. Pollard, C.M.; Meng, X.; Williamson, S.; Dodds, J.; Binns, C.W. Eating out Is Associated with Self-Reported Food Poisoning: A Western Australia Population Perspective, 1998 to 2009. *Public Health Nutr.* **2014**, *17*, 2270–2277, doi:10.1017/S1368980013002371.
89. Ovca, A.; Jevšnik, M.; Jereb, G.; Raspor, P. Effect of Educational Intervention on Young People, Targeting Microbiological Hazards in Domestic Kitchens. *Food Policy* **2016**, *61*, 156–162, doi:10.1016/j.foodpol.2016.03.004.
90. Kilanowski, J.F.; Lin, L. Effects of a Healthy Eating Intervention on Latina Migrant Farmworker

Mothers. *Fam. Community Health* **2013**, *36*, 350–362, doi:10.1097/FCH.0b013e31829d277e.
91. Lievonen, S.; Havulinna, A.S.; Maijala, R. Egg Consumption Patterns and Salmonella Risk in Finland. *J. Food Prot.* **2004**, *67*, 2416–2423, doi:10.4315/0362-028X-67.11.2416.
92. Aydin, G.; Margerison, C.; Worsley, A.; Booth, A. Essential Food and Nutrition Knowledge and Skills for Primary School Children: Australian Parents' Opinions. *HE* **2022**, *122*, 424–439, doi:10.1108/HE-09-2021-0131.
93. Pouillot, R.; Lubran, M.B.; Cates, S.C.; Dennis, S. Estimating Parametric Distributions of Storage Time and Temperature of Ready-to-Eat Foods for U.S. Households. *J. Food Prot.* **2010**, *73*, 312–321, doi:10.4315/0362-028X-73.2.312.
94. Matias, S.L.; Rodriguez-Jordan, J.; McCoin, M. Evaluation of a College-Level Nutrition Course With a Teaching Kitchen Lab. *J. Nutr. Educ. Behav.***2021**, *53*, 787–792, doi:10.1016/j.jneb.2021.02.004.
95. Feng, Y.; Bruhn, C.; Marx, D. Evaluation of Different Food Safety Education Interventions. *Br. Food J.* **2016**, *118*, 762–776, doi:10.1108/BFJ-10-2015-0372.
96. Barrett, T.; Feng, Y. Evaluation of Food Safety Curriculum Effectiveness: A Longitudinal Study of High-School-Aged Youths' Knowledge Retention, Risk-Perception, and Perceived Behavioral Control. *Food Control* **2021**, *121*, 107587, doi:10.1016/j.foodcont.2020.107587.
97. Doménech, E.; Conchado, A.; Escriche, I. Evaluation of Risk Impact of Consumers' Behaviour in Terms of Exposure to Listeria Monocytogenes in Lettuce. *Int J of Food Sci Tech* **2014**, *49*, 2176–2183, doi:10.1111/ijfs.12528.
98. Barrett, T.; Feng, Y.; Chen, H.; Chuang, E.; Feist, S.; Choate, M. Evaluation of the Fight BAC! The Story of Your Dinner Campaign Video: A Multistate Study. *J. Food Prot.* **2020**, *83*, 584–598, doi:10.4315/0362-028X.JFP-19-329.
99. Sanna, A.; Carraro, V.; Sanna, C.; Cabiddu, C.; Brandas, V.; Coroneo, V. Evaluation of the Level of Domestic Hygiene in Household Kitchens. *Ann Ig* **2014**, *26*, 473–481, doi:10.7416/ai.2014.2006.
100. Mullaney, M.I.; Corish, C.A.; Loxley, A. Exploring the Nutrition and Lifestyle Knowledge, Attitudes and Behaviour of Student Home Economics Teachers: Baseline Findings from a 4-year Longitudinal Study. *Int J Consumer Studies* **2008**, *32*, 314–322, doi:10.1111/j.1470-6431.2007.00650.x.
101. Laurenti, P.; De Meo, C.; Sacchini, D.; Spagnolo, A.; Moro, D.; Varacca, A.; Landi, F.; Manes-Gravina, E.; Sgadari, A.; Bernabei, R.; et al. Factors Affecting Safe and Healthy Diet in Older Adults in Italy: Results of a Preliminary Study Performed in a Community-Dwelling Sample. *Public Health Nutr.* **2020**, *23*, 432–438, doi:10.1017/S1368980019002301.
102. Milazzo, A.; Giles, L.C.; Zhang, Y.; Koehler, A.P.; Hiller, J.E.; Bi, P. Factors Influencing Knowledge, Food Safety Practices and Food Preferences During Warm Weather of *Salmonella* and *Campylobacter* Cases in South Australia. *Foodborne Pathog. Dis.* **2017**, *14*, 125–131, doi:10.1089/fpd.2016.2201.
103. Neumark-Sztainer, D.; Hannan, P.J.; Story, M.; Croll, J.; Perry, C. Family Meal Patterns: Associations with Sociodemographic Characteristics and Improved Dietary Intake among Adolescents. *J. Am. Diet. Assoc.* **2003**, *103*, 317–322, doi:10.1053/jada.2003.50048.
104. Wolfson, J.A.; Lahne, J.; Raj, M.; Insolera, N.; Lavelle, F.; Dean, M. Food Agency in the United States: Associations with Cooking Behavior and Dietary Intake. *Nutrients* **2020**, *12*, 877, doi:10.3390/nu12030877.
105. Locher, J.L.; Ritchie, C.S.; Roth, D.L.; Sen, B.; Vickers, K.S.; Vailas, L.I. Food Choice among Homebound Older Adults: Motivations and Perceived Barriers. *J. Nutr. Health Aging* **2009**, *13*, 659–664, doi:10.1007/s12603-009-0194-7.
106. Nesbitt, A.; Majowicz, S.; Finley, R.; Pollari, F.; Pintar, K.; Marshall, B.; Cook, A.; Sargeant, J.; Wilson, J.; Ribble, C.; et al. Food Consumption Patterns in the Waterloo Region, Ontario, Canada: A Cross-Sectional Telephone Survey. *BMC Public Health* **2008**, *8*, 370, doi:10.1186/1471-2458-8-370.
107. Tomaszewska, M.; Trafialek, J.; Suebpongsang, P.; Kolanowski, W. Food Hygiene Knowledge and Practice of Consumers in Poland and in Thailand - A Survey. *Food Control* **2018**, *85*, 76–84, doi:10.1016/j.foodcont.2017.09.022.
108. Mullan, B.; Wong, C.; Todd, J.; Davis, E.; Kothe, E.J. Food Hygiene Knowledge in

Adolescents and Young Adults. *Br. Food J.* **2015**, *117*, 50–61, doi:10.1108/BFJ-03-2013-0060.
109. Thaivalappil, A.; Young, I.; Paco, C.; Jeyapalan, A.; Papadopoulos, A. Food Safety and the Older Consumer: A Systematic Review and Meta-Regression of Their Knowledge and Practices at Home. *Food Control* **2020**, *107*, 106782, doi:10.1016/j.foodcont.2019.106782.
110. Crovato, S.; Pinto, A.; Giardullo, P.; Mascarello, G.; Neresini, F.; Ravarotto, L. Food Safety and Young Consumers: Testing a Serious Game as a Risk Communication Tool. *Food Control* **2016**, *62*, 134–141, doi:10.1016/j.foodcont.2015.10.009.
111. Langiano, E.; Ferrara, M.; Lanni, L.; Viscardi, V.; Abbatecola, A.M.; De Vito, E. Food Safety at Home: Knowledge and Practices of Consumers. *J Public Health* **2012**, *20*, 47–57, doi:10.1007/s10389-011-0437-z.
112. Diplock, K.J.; Jones-Bitton, A.; Leatherdale, S.T.; Rebellato, S.; Hammond, D.; Majowicz, S.E. Food Safety Education Needs of High-School Students: Leftovers, Lunches, and Microwaves. *J. Sch. Health* **2019**, *89*, 578–586, doi:10.1111/josh.12782.
113. Azevedo, I.; Albano, H.; Silva, J.; Teixeira, P. Food Safety in the Domestic Environment. *Food Control* **2014**, *37*, 272–276, doi:10.1016/j.foodcont.2013.09.058.
114. Fischer, A.R.H.; De Jong, A.E.I.; Van Asselt, E.D.; De Jonge, R.; Frewer, L.J.; Nauta, M.J. Food Safety in the Domestic Environment: An Interdisciplinary Investigation of Microbial Hazards During Food Preparation. *Risk Analysis* **2007**, *27*, 1065–1082, doi:10.1111/j.1539-6924.2007.00944.x.
115. Kendall, P.; Scharff, R.; Baker, S.; LeJeune, J.; Sofos, J.; Medeiros, L. Food Safety Instruction Improves Knowledge and Behavior Risk and Protection Factors for Foodborne Illnesses in Pregnant Populations. *Matern. Child Health J.***2017**, *21*, 1686–1698, doi:10.1007/s10995-017-2291-2.
116. Kwon, J.; Wilson, A.N.S.; Bednar, C.; Kennon, L. Food Safety Knowledge and Behaviors of Women, Infant, and Children (WIC) Program Participants in the United States. *J. Food Prot.* **2008**, *71*, 1651–1658, doi:10.4315/0362-028X-71.8.1651.
117. Lazou, T.; Georgiadis, M.; Pentieva, K.; McKevitt, A.; Iossifidou, E. Food Safety Knowledge and Food-Handling Practices of Greek University Students: A Questionnaire-Based Survey. *Food Control* **2012**, *28*, 400–411, doi:10.1016/j.foodcont.2012.05.027.
118. Jevšnik, M.; Ovca, A.; Bauer, M.; Fink, R.; Oder, M.; Sevšek, F. Food Safety Knowledge and Practices among Elderly in Slovenia. *Food Control* **2013**, *31*, 284–290, doi:10.1016/j.foodcont.2012.10.003.
119. Ayaz, W.O.; Priyadarshini, A.; Jaiswal, A.K. Food Safety Knowledge and Practices among Saudi Mothers. *Foods* **2018**, *7*, 193, doi:10.3390/foods7120193.
120. Jevšnik, M.; Česen, A.; Šantić, M.; Ovca, A. Food Safety Knowledge and Practices of Pregnant Women and Postpartum Mothers in Slovenia. *Foods* **2021**, *10*, 2412, doi:10.3390/foods10102412.
121. Evans, E.; Redmond, E. Food Safety Knowledge and Self-Reported Food-Handling Practices in Cancer Treatment. *ONF* **2018**, *45*, E98–E110, doi:10.1188/18.ONF.E98-E110.
122. Bermúdez-Millán, A.; Pérez-Escamilla, R.; Damio, G.; González, A.; Segura-Pérez, S. Food Safety Knowledge, Attitudes, and Behaviors among Puerto Rican Caretakers Living in Hartford, Connecticut. *J. Food Prot.* **2004**, *67*, 512–516, doi:10.4315/0362-028X-67.3.512.
123. Mihalache, O.A.; Dumitraşcu, L.; Nicolau, A.I.; Borda, D. Food Safety Knowledge, Food Shopping Attitude and Safety Kitchen Practices among Romanian Consumers: A Structural Modelling Approach. *Food Control* **2021**, *120*, 107545, doi:10.1016/j.foodcont.2020.107545.
124. Haapala, I.; Probart, C. Food Safety Knowledge, Perceptions, and Behaviors among Middle School Students. *J. Nutr. Educ. Behav.* **2004**, *36*, 71–76, doi:10.1016/S1499-4046(06)60136-X.
125. Sterniša, M.; Smole Možina, S.; Levstek, S.; Kukec, A.; Raspor, P.; Jevšnik, M. Food Safety Knowledge, Self-Reported Practices and Attitude of Poultry Meat Handling among Slovenian Consumers. *Br. Food J.* **2018**, *120*, 1344–1357, doi:10.1108/BFJ-06-2017-0360.
126. Roseman, M.; Kurzynske, J. Food Safety Perceptions and Behaviors of Kentucky Consumers. *J. Food Prot.* **2006**, *69*, 1412–1421, doi:10.4315/0362-028X-69.6.1412.
127. Røssvoll, E.H.; Lavik, R.; Ueland, Ø.; Jacobsen, E.; Hagtvedt, T.; Langsrud, S. Food Safety

Practices among Norwegian Consumers. *J. Food Prot.* **2013**, *76*, 1939–1947, doi:10.4315/0362-028X.JFP-12-269.
128. Trepka, M.J.; Newman, F.L.; Dixon, Z.; Huffman, F.G. Food Safety Practices among Pregnant Women and Mothers in the Women, Infants, and Children Program, Miami, Florida. *J. Food Prot.* **2007**, *70*, 1230–1237, doi:10.4315/0362-028X-70.5.1230.
129. McWilliams, R.M.; Hallman, W.K.; Cuite, C.L.; Senger-Mersich, A.; Sastri, N.; Netterville, L.; Byrd-Bredbenner, C. Food Safety Practices of Homebound Seniors Receiving Home-Delivered Meals. *Top. Clin. Nutr.* **2017**, *32*, 268–281, doi:10.1097/TIN.0000000000000117.
130. Byrd-Bredbenner, C.; Maurer, J.; Wheatley, V.; Schaffner, D.; Bruhn, C.; Blalock, L. Food Safety Self-Reported Behaviors and Cognitions of Young Adults: Results of a National Study. *J. Food Prot.* **2007**, *70*, 1917–1926, doi:10.4315/0362-028X-70.8.1917.
131. Towns, R.E.; Cullen, R.W.; Memken, J.A.; Nnakwe, N.E. Food Safety–Related Refrigeration and Freezer Practices and Attitudes of Consumers in Peoria and Surrounding Counties. *J. Food Prot.* **2006**, *69*, 1640–1645, doi:10.4315/0362-028X-69.7.1640.
132. Kilanowski, J.F.; Moore, L.C. Food Security and Dietary Intake in Midwest Migrant Farmworker Children. *J. Pediatr. Nurs.* **2010**, *25*, 360–366, doi:10.1016/j.pedn.2009.04.008.
133. Carroll, N.; Sadowski, A.; Parizeau, K.; Von Massow, M.; Wallace, A.; Jewell, K.; Ma, D.W.L.; Buchholz, A.C.; Duncan, A.M.; Chan, B.; et al. Food Skills: Associations With Diet Quality and Food Waste Among Canadian Parents. *J. Nutr. Educ. Behav.* **2021**, *53*, 371–379, doi:10.1016/j.jneb.2020.10.018.
134. Mahon, D.; Cowan, C.; Henchion, M.; Fanning, M. FOOD-HANDLING PRACTICES OF IRISH BEEF CONSUMERS. *J. Food Saf.* **2006**, *26*, 72–81, doi:10.1111/j.1745-4565.2005.00028.x.
135. Balzan, S.; Fasolato, L.; Cardazzo, B.; Penon, C.; Novelli, E. Genuine and Natural: The Opinion of Teen Consumers. *Ital J Food Safety* **2017**, *6*, doi:10.4081/ijfs.2017.6183.
136. Taylor, E.V.; Holt, K.G.; Mahon, B.E.; Ayers, T.; Norton, D.; Gould, L.H. Ground Beef Consumption Patterns in the United States, FoodNet, 2006 through 2007. *J. Food Prot.* **2012**, *75*, 341–346, doi:10.4315/0362-028X.JFP-11-333.
137. Mihalache, O.A.; Teixeira, P.; Langsrud, S.; Nicolau, A.I. Hand Hygiene Practices during Meal Preparation—a Ranking among Ten European Countries. *BMC Public Health* **2023**, *23*, 1315, doi:10.1186/s12889-023-16222-5.
138. Nesbitt, A.; Majowicz, S.; Finley, R.; Marshall, B.; Pollari, F.; Sargeant, J.; Ribble, C.; Wilson, J.; Sittler, N. High-Risk Food Consumption and Food Safety Practices in a Canadian Community. *J. Food Prot.* **2009**, *72*, 2575–2586, doi:10.4315/0362-028X-72.12.2575.
139. Parra, P.A.; Kim, H.; Shapiro, M.A.; Gravani, R.B.; Bradley, S.D. Home Food Safety Knowledge, Risk Perception, and Practices among Mexican-Americans. *Food Control* **2014**, *37*, 115–125, doi:10.1016/j.foodcont.2013.08.016.
140. Edfors, E.; Westergren, A. Home-Living Elderly People's Views on Food and Meals. *J. Aging Res.* **2012**, *2012*, 1–9, doi:10.1155/2012/761291.
141. Martins, C.P.C.; Ramos, G.L.P.A.; Pimentel, T.C.; Freitas, M.Q.; Duarte, M.C.K.H.; Azeredo, D.P.R.; Silva, M.C.; Cavalcanti, R.N.; Esmerino, E.A.; Cruz, A.G. How Microwave Technology Is Perceived? A Food Safety Cross-Cultural Study between Brazil and Portugal. *Food Control* **2022**, *134*, 108763, doi:10.1016/j.foodcont.2021.108763.
142. Kennedy, J.; Gibney, S.; Nolan, A.; O'Brien, S.; McMahon, M.A.S.; McDowell, D.; Fanning, S.; Wall, P.G. Identification of Critical Points during Domestic Food Preparation: An Observational Study. *Br. Food J.* **2011**, *113*, 766–783, doi:10.1108/00070701111140106.
143. Henley, S.C.; Stein, S.E.; Quinlan, J.J. Identification of Unique Food Handling Practices That Could Represent Food Safety Risks for Minority Consumers. *J. Food Prot.* **2012**, *75*, 2050–2054, doi:10.4315/0362-028X.JFP-12-146.
144. Jones, L.J.; VanWassenhove-Paetzold, J.; Thomas, K.; Bancroft, C.; Ziatyk, E.Q.; Kim, L.S.-H.; Shirley, A.; Warren, A.C.; Hamilton, L.; George, C.V.; et al. Impact of a Fruit and Vegetable Prescription Program on Health Outcomes and Behaviors in Young Navajo Children. *Curr. Dev.*

Nutr. **2020**, *4*, nzaa109, doi:10.1093/cdn/nzaa109.
145. Ben Hassen, T.; El Bilali, H.; Allahyari, M.S. Impact of COVID-19 on Food Behavior and Consumption in Qatar. *Sustainability* **2020**, *12*, 6973, doi:10.3390/su12176973.
146. Teisl, M.F.; Lando, A.M.; Levy, A.S.; Noblet, C.L. Importance of Cohorts in Analyzing Trends in Safe At-Home Food-Handling Practices. *Food Control* **2016**, *62*, 381–389, doi:10.1016/j.foodcont.2015.10.040.
147. Dharod, J.M.; Pérez-Escamilla, R.; Bermúdez-Millán, A.; Segura-Pérez, S.; Damio, G. Influence of the Fight BAC! Food Safety Campaign on an Urban Latino Population in Connecticut. *J. Nutr. Educ. Behav.* **2004**, *36*, 128–134, doi:10.1016/S1499-4046(06)60149-8.
148. Thaivalappil, A.; Papadopoulos, A.; Young, I. Intentions to Adopt Safe Food Storage Practices in Older Adults: An Application of the Theory of Planned Behaviour. *Br. Food J.***2019**, *122*, 181–197, doi:10.1108/BFJ-07-2019-0483.
149. Carbas, B.; Cardoso, L.; Coelho, A.C. Investigation on the Knowledge Associated with Foodborne Diseases in Consumers of Northeastern Portugal. *Food Control* **2013**, *30*, 54–57, doi:10.1016/j.foodcont.2012.06.028.
150. Slater, J. Is Cooking Dead? The State of H Ome E Conomics F Ood and N Utrition Education in a C Anadian Province. *Int J Consumer Studies* **2013**, *37*, 617–624, doi:10.1111/ijcs.12042.
151. Møretrø, T.; Martens, L.; Teixeira, P.; Ferreira, V.B.; Maia, R.; Maugesten, T.; Langsrud, S. Is Visual Motivation for Cleaning Surfaces in the Kitchen Consistent with a Hygienically Clean Environment? *Food Control* **2020**, *111*, 107077, doi:10.1016/j.foodcont.2019.107077.
152. Mihalache, O.A.; Møretrø, T.; Borda, D.; Dumitraşcu, L.; Neagu, C.; Nguyen-The, C.; Maître, I.; Didier, P.; Teixeira, P.; Lopes Junqueira, L.O.; et al. Kitchen Layouts and Consumers' Food Hygiene Practices: Ergonomics versus Safety. *Food Control* **2022**, *131*, 108433, doi:10.1016/j.foodcont.2021.108433.
153. Muñoz, K.; Wagner, M.; Pauli, F.; Christ, J.; Reese, G. Knowledge and Behavioral Habits to Reduce Mycotoxin Dietary Exposure at Household Level in a Cohort of German University Students. *Toxins* **2021**, *13*, 760, doi:10.3390/toxins13110760.
154. Lavelle, F.; Spence, M.; Hollywood, L.; McGowan, L.; Surgenor, D.; McCloat, A.; Mooney, E.; Caraher, M.; Raats, M.; Dean, M. Learning Cooking Skills at Different Ages: A Cross-Sectional Study. *Int J Behav Nutr Phys Act* **2016**, *13*, 119, doi:10.1186/s12966-016-0446-y.
155. Bondarianzadeh, D.; Yeatman, H.; Condon-Paoloni, D. Listeria Education in Pregnancy: Lost Opportunity for Health Professionals. *Aust. N. Z. J. Public Health* **2007**, *31*, 468–474, doi:10.1111/j.1753-6405.2007.00120.x.
156. Ovca, A.; Jevšnik, M. Maintaining a Cold Chain from Purchase to the Home and at Home: Consumer Opinions. *Food Control* **2009**, *20*, 167–172, doi:10.1016/j.foodcont.2008.03.010.
157. Jeong, G.; Park, S.W.; Lee, Y.K.; Ko, S.Y.; Shin, S.M. Maternal Food Restrictions during Breastfeeding. *Korean J Pediatr* **2017**, *60*, 70, doi:10.3345/kjp.2017.60.3.70.
158. García-Esquinas, E.; Pérez-Gómez, B.; Fernández, M.A.; Pérez-Meixeira, A.M.; Gil, E.; Paz, C.D.; Iriso, A.; Sanz, J.C.; Astray, J.; Cisneros, M.; et al. Mercury, Lead and Cadmium in Human Milk in Relation to Diet, Lifestyle Habits and Sociodemographic Variables in Madrid (Spain). *Chemosphere* **2011**, *85*, 268–276, doi:10.1016/j.chemosphere.2011.05.029.
159. Osaili, T.M.; Obaid, R.S.; Alowais, K.; Almahmood, R.; Almansoori, M.; Alayadhi, N.; Alowais, N.; Waheed, K.; Dhanasekaran, D.K.; Al-Nabulsi, A.A.; et al. Microbiological Quality of Kitchens Sponges Used in University Student Dormitories. *BMC Public Health* **2020**, *20*, 1322, doi:10.1186/s12889-020-09452-4.
160. Quandt, S.A.; Groeschel-Johnson, A.; Kinzer, H.T.; Jensen, A.; Miles, K.; O'Hara, H.M.; Chen, H.; Arcury, T.A. Migrant Farmworker Nutritional Strategies: Implications for Diabetes Management. *J. Agromedicine* **2018**, *23*, 347–354, doi:10.1080/1059924X.2018.1501453.
161. Simonds, K.; Zhang, L.Y.; Matthews, J.I. " *My Roommates Would Laugh at Me* ": Young Males Reveal Embarrassment over Lack of Food Skills. *Can. J. Diet. Pract. Res.* **2021**, *82*, 51–58, doi:10.3148/cjdpr-2020-033.

162. Brombin, A.; Mascarello, G.; Pinto, A.; Crovato, S.; Ricaldi, G.; Giaretta, M.; Ravarotto, L. New Ways of Spreading Food Safety Online: The Role of Food Bloggers in Risk Communication. *Br. Food J.***2022**, *124*, 775–794, doi:10.1108/BFJ-01-2021-0044.
163. Nutritional Self-Management of Elderly Widows in Rural Communities. *The Gerontologist* **2000**, *40*, 86–96, doi:10.1093/geront/40.1.86.
164. Kendall, P.A.; Elsbernd, A.; Sinclair, K.; Schroeder, M.; Chen, G.; Bergmann, V.; Hillers, V.N.; Medeiros, L.C. Observation Versus Self-Report: Validation of a Consumer Food Behavior Questionnaire. *J. Food Prot.* **2004**, *67*, 2578–2586, doi:10.4315/0362-028X-67.11.2578.
165. Shumaker, E.T.; Kirchner, M.; Cates, S.C.; Shelley, L.; Goulter, R.; Goodson, L.; Bernstein, C.; Lavallee, A.; Jaykus, L.-A.; Chapman, B. Observational Study of the Impact of a Food Safety Intervention on Consumer Poultry Washing. *J. Food Prot.* **2022**, *85*, 615–625, doi:10.4315/JFP-21-397.
166. Hoelzl, C.; Mayerhofer, U.; Steininger, M.; Brüller, W.; Hofstädter, D.; Aldrian, U. Observational Trial of Safe Food Handling Behavior during Food Preparation Using the Example of Campylobacter Spp. *J. Food Prot.* **2013**, *76*, 482–489, doi:10.4315/0362-028X.JFP-12-231.
167. Evans, E.W.; Redmond, E.C. Older Adult Consumer Knowledge, Attitudes, and Self-Reported Storage Practices of Ready-to-Eat Food Products and Risks Associated with Listeriosis. *J. Food Prot.* **2016**, *79*, 263–272, doi:10.4315/0362-028X.JFP-15-312.
168. Evans, E.W.; Redmond, E.C. Older Adult Consumers' Attitudes and Perceptions of Risk, Control, and Responsibility for Food Safety in the Domestic Kitchen. *J. Food Prot.* **2019**, *82*, 371–378, doi:10.4315/0362-028X.JFP-18-357.
169. Kosa, K.M.; Cates, S.C.; Brophy, J.; Godwin, S.; Chambers, D.; Iv, E.C. Older Adults and Parents of Young Children Have Different Handling Practices for Raw Poultry. *J. Food Prot.* **2019**, *82*, 200–206, doi:10.4315/0362-028X.JFP-18-323.
170. Au, L.E.; Whaley, S.; Rosen, N.J.; Meza, M.; Ritchie, L.D. Online and In-Person Nutrition Education Improves Breakfast Knowledge, Attitudes, and Behaviors: A Randomized Trial of Participants in the Special Supplemental Nutrition Program for Women, Infants, and Children. *J. Acad. Nutr. Diet.* **2016**, *116*, 490–500, doi:10.1016/j.jand.2015.10.012.
171. Kasza, G.; Csenki, E.Z.; Izsó, T.; Scholderer, J. Paradoxical Risk Mitigation Behavior in Private Households. *Food Control* **2022**, *138*, 109032, doi:10.1016/j.foodcont.2022.109032.
172. Marília Prada; Saraiva, M.; Godinho, C.A.; Tourais, B.; Cavalheiro, B.P.; Garrido, M.V. Parental Perceptions and Practices Regarding Sugar Intake by School-Aged Children: A Qualitative Study with Portuguese Parents. *Appetite* **2021**, *166*, 105471, doi:10.1016/j.appet.2021.105471.
173. Hawthorne, D.L.; Neilson, L.J.; Macaskill, L.A.; Luk, J.M.H.; Horner, E.J.; Parks, C.A.; Salvadori, M.I.; Seabrook, J.A.; Dworatzek, P.D.N. Parental Reports of Lunch-Packing Behaviours Lack Accuracy: Reported Barriers and Facilitators to Packing School Lunches. *Can. J. Diet. Pract. Res.* **2018**, *79*, 99–105, doi:10.3148/cjdpr-2018-011.
174. Worsley, A.; Wang, W.C.; Byrne, S.; Yeatman, H. Patterns of Food Safety Knowledge among Australians: A Latent Class Approach. *J. Food Prot.* **2013**, *76*, 646–652, doi:10.4315/0362-028X.JFP-12-449.
175. Harshman, S.G.; Castro, I.; Perkins, M.; Luo, M.; Barrett Mueller, K.; Cena, H.; Portale, S.; Raspini, B.; Taveras, E.; Fiechtner, L. Pediatric Weight Management Interventions Improve Prevalence of Overeating Behaviors. *Int J Obes* **2022**, *46*, 630–636, doi:10.1038/s41366-021-00989-x.
176. Park, S.; Onufrak, S.; Patel, A.; Sharkey, J.R.; Blanck, H.M. Perceptions of Drinking Water Safety and Their Associations with Plain Water Intake among US Hispanic Adults. *J. Water Health* **2019**, *17*, 587–596, doi:10.2166/wh.2019.015.
177. Millman, C.; Rigby, D.; Edward-Jones, G.; Lighton, L.; Jones, D. Perceptions, Behaviours and Kitchen Hygiene of People Who Have and Have Not Suffered Campylobacteriosis: A Case Control Study. *Food Control* **2014**, *41*, 82–90, doi:10.1016/j.foodcont.2014.01.002.
178. Ma, J.; Almanza, B.A.; Ge, L.; Her, E.; Liu, Y.; Lando, A.; Wu, F.; Verrill, L. Pet Ownership and Pet Type Influence Food Safety in the Home: Evidence from a National Survey. *J. Food Prot.* **2020**, *83*, 1553–1560, doi:10.4315/JFP-20-057.

179. Leonard, D.; Aquino, D.; Hadgraft, N.; Thompson, F.; Marley, J.V. Poor Nutrition from First Foods: A Cross-sectional Study of Complementary Feeding of Infants and Young Children in Six Remote Aboriginal Communities across Northern Australia. *Nutr. Diet.***2017**, *74*, 436–445, doi:10.1111/1747-0080.12386.
180. Bearth, A.; Cousin, M.-E.; Siegrist, M. Poultry Consumers' Behaviour, Risk Perception and Knowledge Related to Campylobacteriosis and Domestic Food Safety. *Food Control* **2014**, *44*, 166–176, doi:10.1016/j.foodcont.2014.03.055.
181. Tendero, A.; Bernabéu, R. Preference Structure for Cheese Consumers: A Spanish Case Study. *Br. Food J.* **2005**, *107*, 60–73, doi:10.1108/00070700510579144.
182. Cater, M.; Gravois, R.; Guerra Gaitan, G.G.; Xu, W. Pregnant Women's Confidence and Perceptions on Practices Related to Food Safety: A Study in Louisiana. *Food Control* **2020**, *113*, 107175, doi:10.1016/j.foodcont.2020.107175.
183. Shiferaw, B.; Yang, S.; Cieslak, P.; Vugia, D.; Marcus, R.; Koehler, J.; Deneen, V.; Angulo, F. Prevalence of High-Risk Food Consumption and Food-Handling Practices among Adults: A Multistate Survey, 1996 to 1997. *J. Food Prot.* **2000**, *63*, 1538–1543, doi:10.4315/0362-028X-63.11.1538.
184. Jakobsen, L.S.; Georgiadis, S.; Nielsen, B.F.; Bokkers, B.G.H.; Boriani, E.; Duedahl-Olesen, L.; Hald, T.; Nauta, M.J.; Stockmarr, A.; Pires, S.M. Probabilistic Approach for Assessing Cancer Risk Due to Benzo[a]Pyrene in Barbecued Meat: Informing Advice for Population Groups. *PLoS ONE* **2018**, *13*, e0207032, doi:10.1371/journal.pone.0207032.
185. Byrd-Bredbenner, C.; Martin-Biggers, J.; Povis, G.A.; Worobey, J.; Hongu, N.; Quick, V. Promoting Healthy Home Environments and Lifestyles in Families with Preschool Children: HomeStyles, a Randomized Controlled Trial. *Contemp. Clin. Trials* **2018**, *64*, 139–151, doi:10.1016/j.cct.2017.10.012.
186. Van Velsen, L.; Beaujean, D.J.; Van Gemert-Pijnen, J.E.; Van Steenbergen, J.E.; Timen, A. Public Knowledge and Preventive Behavior during a Large-Scale Salmonella Outbreak: Results from an Online Survey in the Netherlands. *BMC Public Health* **2014**, *14*, 100, doi:10.1186/1471-2458-14-100.
187. Steelfisher, G.; Weldon, K.; Benson, J.M.; Blendon, R.J. PUBLIC PERCEPTIONS OF FOOD RECALLS AND PRODUCTION SAFETY: TWO SURVEYS OF THE AMERICAN PUBLIC. *J. Food Saf.* **2010**, *30*, 848–866, doi:10.1111/j.1745-4565.2010.00246.x.
188. Crovato, S.; Pinto, A.; Di Martino, G.; Mascarello, G.; Rizzoli, V.; Marcolin, S.; Ravarotto, L. Purchasing Habits, Sustainability Perceptions, and Welfare Concerns of Italian Consumers Regarding Rabbit Meat. *Foods* **2022**, *11*, 1205, doi:10.3390/foods11091205.
189. Litman, L.; Williams, M.T.; Rosen, Z.; Weinberger-Litman, S.L.; Robinson, J. Racial Disparities in Cleanliness Attitudes Mediate Purchasing Attitudes Toward Cleaning Products: A Serial Mediation Model. *J. Racial Ethn. Health Disparities* **2018**, *5*, 838–846, doi:10.1007/s40615-017-0429-y.
190. Ravarotto, L.; Crovato, S.; Mantovani, C.; D'Este, F.; Pinto, A.; Mascarello, G. Reducing Microbiological Risk in the Kitchen: Piloting Consensus Conference Methodology as a Communication Strategy. *Journal of Risk Research* **2016**, *19*, 934–950, doi:10.1080/13669877.2015.1017828.
191. Garayoa, R.; Córdoba, M.; García-Jalón, I.; Sanchez-Villegas, A.; Vitas, A.I. Relationship between Consumer Food Safety Knowledge and Reported Behavior among Students from Health Sciences in One Region of Spain. *J. Food Prot.* **2005**, *68*, 2631–2636, doi:10.4315/0362-028X-68.12.2631.
192. Fischer, A.R.H.; Frewer, L.J. Reliability of the Rasch Food Safety Practices Scale. *Appetite* **2009**, *53*, 241–244, doi:10.1016/j.appet.2009.02.018.
193. Parry, S.M.; Palmer, S.R.; Slader, J.; Humphrey, T.; The South East Wales Infectious Disease Liaison Group Risk Factors for Salmonella Food Poisoning in the Domestic Kitchen – a Case Control Study. *Epidemiol. Infect.* **2002**, *129*, 277–285, doi:10.1017/S0950268802007331.
194. Evans, K.S.; Teisl, M.F.; Lando, Amy.M.; Liu, S.T. Risk Perceptions and Food-Handling Practices in the Home. *Food Policy* **2020**, *95*, 101939, doi:10.1016/j.foodpol.2020.101939.

195. Obande, D.; Young, I. Safe Food Refrigeration Knowledge, Attitudes, and Practices of University Students. *Br. Food J.* **2020**, *122*, 1085–1098, doi:10.1108/BFJ-05-2019-0327.
196. Almli, V.L.; Galler, M.; Møretrø, T.; Langsrud, S.; Gaarder, M.Ø.; Ueland, Ø. Safe Week, Unsafe Weekend? Consumers' Self-Reported Food Safety Practices and Stomach Sickness in Cabin Environments of Varying Infrastructure Levels. *Food Control* **2022**, *142*, 109215, doi:10.1016/j.foodcont.2022.109215.
197. Li-Cohen, A.E.; Bruhn, C.M. Safety of Consumer Handling of Fresh Produce from the Time of Purchase to the Plate: A Comprehensive Consumer Survey. *J. Food Prot.* **2002**, *65*, 1287–1296, doi:10.4315/0362-028X-65.8.1287.
198. Kurtz, A.; Thomopoulos, R. Safety vs. Sustainability Concerns of Infant Food Users: French Results and European Perspectives. *Sustainability* **2021**, *13*, 10074, doi:10.3390/su131810074.
199. Mol, S.; Akay, K.U.; Guney, G.Ç. Seafood Safety at Home: Knowledge and Practices. *Int. J. Gastron. Food Sci.* **2018**, *13*, 95–100, doi:10.1016/j.ijgfs.2018.07.003.
200. Lange, M.; Göranzon, H.; Marklinder, I. Self-Reported Food Safety Knowledge and Behaviour among Home and Consumer Studies Students. *Food Control* **2016**, *67*, 265–272, doi:10.1016/j.foodcont.2016.03.014.
201. Ali, M.M.; Verrill, L.; Zhang, Y. Self-Reported Hand Washing Behaviors and Foodborne Illness: A Propensity Score Matching Approach. *J. Food Prot.* **2014**, *77*, 352–358, doi:10.4315/0362-028X.JFP-13-286.
202. Junqueira, L.; Truninger, M.; Almli, V.L.; Ferreira, V.; Maia, R.L.; Teixeira, P. Self-Reported Practices by Portuguese Consumers Regarding Eggs' Safety: An Analysis Based on Critical Consumer Handling Points. *Food Control* **2022**, *133*, 108635, doi:10.1016/j.foodcont.2021.108635.
203. DeDonder, S.; Jacob, C.J.; Surgeoner, B.V.; Chapman, B.; Phebus, R.; Powell, D.A. Self-reported and Observed Behavior of Primary Meal Preparers and Adolescents during Preparation of Frozen, Uncooked, Breaded Chicken Products. *Br. Food J.* **2009**, *111*, 915–929, doi:10.1108/00070700910992844.
204. Vadiveloo, M.K.; Parker, H.W.; Juul, F.; Parekh, N. Sociodemographic Differences in the Dietary Quality of Food-at-Home Acquisitions and Purchases among Participants in the U.S. Nationally Representative Food Acquisition and Purchase Survey (FoodAPS). *Nutrients* **2020**, *12*, 2354, doi:10.3390/nu12082354.
205. Rettenbacher-Riefler, S.; Ziehm, D.; Kreienbrock, L.; Campe, A.; Pulz, M.; Dreesman, J. Sporadic Salmonellosis in Lower Saxony, Germany, 2011–2013: Raw Ground Pork Consumption Is Associated with *Salmonella* Typhimurium Infections and Foreign Travel with *Salmonella* Enteritidis Infections. *Epidemiol. Infect.* **2015**, *143*, 2777–2785, doi:10.1017/S0950268814003768.
206. Al-Shabib, N.A.; Husain, F.M.; Khan, J.M. Study on Food Safety Concerns, Knowledge and Practices among University Students in Saudi Arabia. *Food Control* **2017**, *73*, 202–208, doi:10.1016/j.foodcont.2016.08.005.
207. Sampers, I.; Berkvens, D.; Jacxsens, L.; Ciocci, M.-C.; Dumoulin, A.; Uyttendaele, M. Survey of Belgian Consumption Patterns and Consumer Behaviour of Poultry Meat to Provide Insight in Risk Factors for Campylobacteriosis. *Food Control* **2012**, *26*, 293–299, doi:10.1016/j.foodcont.2012.01.054.
208. Gilbert, S.E.; Whyte, R.; Bayne, G.; Paulin, S.M.; Lake, R.J.; Van Der Logt, P. Survey of Domestic Food Handling Practices in New Zealand. *Int. J. Food Microbiol.* **2007**, *117*, 306–311, doi:10.1016/j.ijfoodmicro.2007.05.004.
209. Migliorati, G.; Prencipe, V.A.; Iannetti, L.; Di Giannatale, E.; Matteucci, O.; Salini, R.; Calistri, P. Survey of Domestic Food Purchases and Related Home Handling Practices in the Abruzzo Region (Central Italy): Data Collection and Analysis through a Language-Independent Classification System. *Food Control* **2015**, *49*, 23–33, doi:10.1016/j.foodcont.2013.09.008.
210. Al-Sakkaf, A.; Redmond, E.; Brennan, C.; Gooneratne, R. Survey of New Zealand Poultry Consumers' Handling of Raw Poultry and Food Safety Awareness To Provide Insight into Risk Factors for Campylobacteriosis. *J. Food Prot.* **2021**, *84*, 1640–1647, doi:10.4315/JFP-21-034.

211. Carrasco, E.; Pérez-Rodríguez, F.; Valero, A.; García-Gimeno, R.M.; Zurera, G. Survey of Temperature and Consumption Patterns of Fresh-Cut Leafy Green Salads: Risk Factors for Listeriosis. *J. Food Prot.* **2007**, *70*, 2407–2412, doi:10.4315/0362-028X-70.10.2407.
212. Wucher, H.; Klingshirn, A.; Brugger, L.; Stamminger, R.; Geppert, J.; Kölzer, B.; Engstler, A.; Härlen, J. Tackling Food Waste: Impact of German Consumer Behaviour on Food in Chilled Storage. *Foods* **2020**, *9*, 1462, doi:10.3390/foods9101462.
213. Hagedorn, R.L.; White, J.A.; Franzen-Castle, L.; Colby, S.E.; Kattelmann, K.K.; White, A.A.; Olfert, M.D. Teens Implementing a Childhood Obesity Prevention Program in the Community: Feasibility and Perceptions of a Partnership with HSTA and iCook 4-H. *IJERPH* **2018**, *15*, 934, doi:10.3390/ijerph15050934.
214. Andritsos, N.D.; Stasinou, V.; Tserolas, D.; Giaouris, E. Temperature Distribution and Hygienic Status of Domestic Refrigerators in Lemnos Island, Greece. *Food Control* **2021**, *127*, 108121, doi:10.1016/j.foodcont.2021.108121.
215. Taylor, A.W.; Coveney, J.; Ward, P.R.; Dal Grande, E.; Mamerow, L.; Henderson, J.; Meyer, S.B. The Australian Food and Trust Survey: Demographic Indicators Associated with Food Safety and Quality Concerns. *Food Control* **2012**, *25*, 476–483, doi:10.1016/j.foodcont.2011.11.003.
216. Menini, A.; Mascarello, G.; Giaretta, M.; Brombin, A.; Marcolin, S.; Personeni, F.; Pinto, A.; Crovato, S. The Critical Role of Consumers in the Prevention of Foodborne Diseases: An Ethnographic Study of Italian Families. *Foods* **2022**, *11*, 1006, doi:10.3390/foods11071006.
217. Røssvoll, E.; Langsrud, S.; Bloomfield, S.; Moen, B.; Heir, E.; Møretrø, T. The Effects of Different Hygiene Procedures in Reducing Bacterial Contamination in a Model Domestic Kitchen. *J Appl Microbiol* **2015**, *119*, 582–593, doi:10.1111/jam.12869.
218. Lee, H.-J. The Impact of Consumer Competence in Purchasing Foods on Satisfaction with Food-Related Consumer Policies and Satisfaction with Food-Related Life through Perceptions of Food Safety. *Foods* **2020**, *9*, 1103, doi:10.3390/foods9081103.
219. AlTarrah, D.; AlShami, E.; AlHamad, N.; AlBesher, F.; Devarajan, S. The Impact of Coronavirus COVID-19 Pandemic on Food Purchasing, Eating Behavior, and Perception of Food Safety in Kuwait. *Sustainability* **2021**, *13*, 8987, doi:10.3390/su13168987.
220. McGowan, L.; Pot, G.K.; Stephen, A.M.; Lavelle, F.; Spence, M.; Raats, M.; Hollywood, L.; McDowell, D.; McCloat, A.; Mooney, E.; et al. The Influence of Socio-Demographic, Psychological and Knowledge-Related Variables alongside Perceived Cooking and Food Skills Abilities in the Prediction of Diet Quality in Adults: A Nationally Representative Cross-Sectional Study. *Int J Behav Nutr Phys Act* **2016**, *13*, 111, doi:10.1186/s12966-016-0440-4.
221. Vegara, A.; Rita Festino, A.; Di Ciccio, P.; Costanzo, C.; Pennisi, L.; Ianieri, A. The Management of the Domestic Refrigeration: Microbiological Status and Temperature. *Br. Food J.* **2014**, *116*, 1047–1057, doi:10.1108/BFJ-05-2012-0103.
222. Mascarello, G.; Pinto, A.; Parise, N.; Crovato, S.; Ravarotto, L. The Perception of Food Quality. Profiling Italian Consumers. *Appetite* **2015**, *89*, 175–182, doi:10.1016/j.appet.2015.02.014.
223. Anderson, H.A.; Hanrahan, L.P.; Smith, A.; Draheim, L.; Kanarek, M.; Olsen, J. The Role of Sport-Fish Consumption Advisories in Mercury Risk Communication: A 1998–1999 12-State Survey of Women Age 18–45. *Environ. Res.* **2004**, *95*, 315–324, doi:10.1016/j.envres.2004.01.004.
224. Fulkerson, J.A.; Nelson, M.C.; Lytle, L.; Moe, S.; Heitzler, C.; Pasch, K.E. The Validation of a Home Food Inventory. *Int J Behav Nutr Phys Act* **2008**, *5*, 55, doi:10.1186/1479-5868-5-55.
225. Dumitrașcu, L.; Nicolau, A.I.; Neagu, C.; Didier, P.; Maître, I.; Nguyen-The, C.; Skuland, S.E.; Møretrø, T.; Langsrud, S.; Truninger, M.; et al. Time-Temperature Profiles and Listeria Monocytogenes Presence in Refrigerators from Households with Vulnerable Consumers. *Food Control* **2020**, *111*, 107078, doi:10.1016/j.foodcont.2019.107078.
226. Butcher, L.M.; O'Sullivan, T.A.; Ryan, M.M.; Lo, J.; Nyanjom, J.; Wilkins, H.C.; Devine, A. To Dine in or Not to Dine in: A Comparison of Food Selection and Preparation Behaviours in Those with and without Food Security. *Health Prom J of Aust* **2021**, *32*, 267–282, doi:10.1002/hpja.427.
227. Appelhans, B.M.; French, S.A.; Tangney, C.C.; Powell, L.M.; Wang, Y. To What Extent Do

Food Purchases Reflect Shoppers' Diet Quality and Nutrient Intake? *Int J Behav Nutr Phys Act* **2017**, *14*, 46, doi:10.1186/s12966-017-0502-2.
228. Fischer, A.R.H.; Frewer, L.J.; Nauta, M.J. Toward Improving Food Safety in the Domestic Environment: A Multi-Item Rasch Scale for the Measurement of the Safety Efficacy of Domestic Food-Handling Practices. *Risk Anal.* **2006**, *26*, 1323–1338, doi:10.1111/j.1539-6924.2006.00813.x.
229. Li, S.; Kallas, Z.; Rahmani, D.; Gil, J.M. Trends in Food Preferences and Sustainable Behavior during the COVID-19 Lockdown: Evidence from Spanish Consumers. *Foods* **2021**, *10*, 1898, doi:10.3390/foods10081898.
230. Lando, A.M.; Chen, C.C. Trends in Ownership and Usage of Food Thermometers in the United States, 1998 through 2010. *J. Food Prot.* **2012**, *75*, 556–562, doi:10.4315/0362-028X.JFP-11-314.
231. Fein, S.B.; Lando, A.M.; Levy, A.S.; Teisl, M.F.; Noblet, C. Trends in U.S. Consumers' Safe Handling and Consumption of Food and Their Risk Perceptions, 1988 through 2010. *J. Food Prot.* **2011**, *74*, 1513–1523, doi:10.4315/0362-028X.JFP-11-017.
232. Swinehart, M.; Harris, L.J.; Anderson, N.M.; Feng, Y. U.S. Consumer Practices of Homemade Nut-Based Dairy Analogs and Soaked Nuts. *J. Food Prot.* **2023**, *86*, 100132, doi:10.1016/j.jfp.2023.100132.
233. Van Boxstael, S.; Devlieghere, F.; Berkvens, D.; Vermeulen, A.; Uyttendaele, M. Understanding and Attitude Regarding the Shelf Life Labels and Dates on Pre-Packed Food Products by Belgian Consumers. *Food Control* **2014**, *37*, 85–92, doi:10.1016/j.foodcont.2013.08.043.
234. Freivogel, C.; H. M. Visschers, V. Understanding the Underlying Psychosocial Determinants of Safe Food Handling among Consumers to Mitigate the Transmission Risk of Antimicrobial-Resistant Bacteria. *IJERPH* **2020**, *17*, 2546, doi:10.3390/ijerph17072546.
235. Yuan, J.J.; Yi, S.; Williams, H.A.; Park, O.-H. US Consumers' Perceptions of Imperfect "Ugly" Produce. *Br. Food J.***2019**, *121*, 2666–2682, doi:10.1108/BFJ-03-2019-0206.
236. Borda, D.; Mihalache, O.A.; Nicolau, A.I.; Teixeira, P.; Langsrud, S.; Dumitrascu, L. Using Tactile Cold Perceptions as an Indicator of Food Safety-a Hazardous Choice. *Food Control* **2020**, *111*, 107069, doi:10.1016/j.foodcont.2019.107069.
237. Didier, P.; Nguyen-The, C.; Martens, L.; Foden, M.; Dumitrascu, L.; Mihalache, A.O.; Nicolau, A.I.; Skuland, S.E.; Truninger, M.; Junqueira, L.; et al. Washing Hands and Risk of Cross-Contamination during Chicken Preparation among Domestic Practitioners in Five European Countries. *Food Control* **2021**, *127*, 108062, doi:10.1016/j.foodcont.2021.108062.
238. Gauci, C.; Gauci, A.A. What Does the Food Handler in the Home Know about Salmonellosis and Food Safety? *J. R. Soc. Promot. Health* **2005**, *125*, 136–142, doi:10.1177/146642400512500318.
239. Brennan, M.; McCarthy, M.; Ritson, C. Why Do Consumers Deviate from Best Microbiological Food Safety Advice? An Examination of 'High-Risk' Consumers on the Island of Ireland. *Appetite* **2007**, *49*, 405–418, doi:10.1016/j.appet.2006.12.006.
240. Chuang, E.; Thomas, M.; Feng, Y. Young Adult Food Safety Knowledge Gaps and Perceptions of Roommates' Food Handling Practices: A Survey of University Students in Indiana. *Food Control* **2021**, *126*, 108055, doi:10.1016/j.foodcont.2021.108055.
241. Mazengia, E.; Fisk, C.; Liao, G.; Huang, H.; Meschke, J.S. Direct Observational Study of the Risk of Cross-Contamination during Raw Poultry Handling: Practices in Private Homes. *Food Prot. Trends.* 2015, 35, 8–23.
242. Bruhn, C.M. Chicken Preparation in the Home: An Observational Study. *Food Prot. Trends.* 2014, 34, 318–330.
243. Green, L.R.; Selman, C.A.Factors Impacting Food Workers' and Managers' Safe Food Preparation Practices: A Qualitative Study. *J. Environ. Health* 2007, 70, 13–21.
244. Scott, E.; Herbold, N. An In-Home Video Study and Questionnaire Survey of Food Preparation, Kitchen Sanitation, and Hand Washing Practices. *J. Environ. Health* 2010, 72, 8–13.
245. Byrd-Bredbenner, C.; Santiago, E.; Eck, K.M.; Delaney, C.L.; Quick, V.M.; Pozzoli, A.; Worobey, J.; Shelnutt, K.P.; Olfert, M.D. HomeStyles-2: Randomized Controlled Trial Protocol for a Web-Based Obesity Prevention Program for Families with Children in Middle Childhood. *Contemp.*

Clin. Trials 2022, 112, 106644. https://doi.org/10.1016/j.cct.2021.106644
246. Petljak, K.; Štulec, I.; Renko, S. Consumers' Willingness to Pay More for Organic Food in Croatia. *Ekonomski vjesnik* 2017, 30, 441–455.
247. Gharbi, A.; Doerflinger, F.; Sanaa, M.; et al. Food Handling Practices and Associated Risk Factors for Foodborne Illnesses: A Systematic Review. *Int. J. Food Microbiol.* 2021, 338, 108983.

5.2 Grey literature (27 documents)

1. Ambrus, Á.; Horváth, Z.; Farkas, Z.; Dorogházi, E.; Cseh, J.; Petrova, S.; Dimitrov, P.; Duleva, V.; Rangelova, L.; Chikova-Iscener, E.; et al. Pilot Study in the View of a Pan-European Dietary Survey – Adolescents, Adults and Elderly. *EFSA Support. Publ.* 2013, *10*, EN-508. https://doi.org/10.2903/sp.efsa.2013.EN-508
2. Hadjicharalambous, C.; Grispoldi, L.; Goga, B.C. Quantitative Risk Assessment of *Listeria monocytogenes* in a Traditional RTE Product. *EFSA Support. Publ.* 2019, *17*, EN-906. https://doi.org/10.2903/j.efsa.2019.e170906
3. Popovska, S.; Jonovska, K.; Adzija, S.S.; Mickova, S.T.; Spiroski, I. National Dietary Survey on the Children Population in the Republic of North Macedonia. *EFSA Support. Publ.* 2022, *19*, EN-7169. https://doi.org/10.2903/sp.efsa.2022.EN-7169
4. ICF; McEntaggart, K.; Chirico, S.; Etienne, J.; Rigoni, M.; Papoutsis, S.; Leather, J. EU Insights: Chemical Mixtures Awareness, Understanding and Risk Perceptions. *EFSA Support. Publ.* 2019, *16*, EN-1602. https://doi.org/10.2903/sp.efsa.2019.EN-1602
5. ICF (in association with GfK); Etienne, J.; Chirico, S.; McEntaggart, K.; Papoutsis, S.; Millstone, E. EU Insights – Consumer Perceptions of Emerging Risks in the Food Chain. *EFSA Support. Publ.* 2018, *15*, EN-1394. https://doi.org/10.2903/sp.efsa.2018.EN-1394
6. Kaartinen, N.; Tapanainen, H.; Reinivuo, H.; Pakkala, H.; Aalto, S.; Raulio, S.; Männistö, S.; Korhonen, T.; Virtanen, S.; Borodulin, K.; et al. The Finnish National Dietary Survey in Adults and Elderly (FinDiet 2017). *EFSA Support. Publ.* 2020, *17*, EN-1914. https://doi.org/10.2903/sp.efsa.2020.EN-1914
7. Lopes, C.; Torres, D.; Oliveira, A.; Severo, M.; Guiomar, S.; Alarcão, V.; Vilela, S.; Ramos, E.; Rodrigues, S.; Oliveira, L.; et al. National Food, Nutrition and Physical Activity Survey of the Portuguese General Population. *EFSA Support. Publ.*2017, *14*, EN-1341. https://doi.org/10.2903/sp.efsa.2017.EN-1341
8. National Institute for Public Health and the Environment (RIVM); van Rossum, C.; Nelis, K.; Wilson, C.; Ocké, M. National Dietary Survey in 2012-2016 on the General Population Aged 1–79 Years in the Netherlands. *EFSA Support. Publ.* 2018, *15*, EN-1488. https://doi.org/10.2903/sp.efsa.2018.EN-1488
9. Neagu, M.; Nicolescu, F.; Tănăsescu, B.; Stan, M.; Zugravu, C.; Cucu, A.; Galan, A.; Partin, A. Romanian National Food Consumption Survey for Adolescents, Adults and Elderly. *EFSA Support. Publ.* 2020, *17*, EN-1923. https://doi.org/10.2903/sp.efsa.2020.EN-1923
10. Tsigarida, E.; Gaitis, F.; Garofalakis, G.; Papanastasiou, D.; Marakis, G.; Mila, S.; Barberis, K.; Tzoumanika, F.; Aspridou, Z.; Tsaloumi, S.; et al. Evaluation of Listeriosis Risk Related with the Consumption of Non-Prepackaged Ready-to-Eat Cooked Meat Products Handled at Retail Stores in Greece. *EFSA Support. Publ.* 2019, *16*, EN-1677. https://doi.org/10.2903/sp.efsa.2019.EN-1677
11. Zucconi, S.; Volpato, C.; Adinolfi, F.; Gandini, E.; Gentile, E.; Loi, A.; Fioriti, L. Gathering Consumption Data on Specific Consumer Groups of Energy Drinks. *EFSA Support. Publ.* 2013, *10*, EN-394. https://doi.org/10.2903/sp.efsa.2013.EN-394
12. Lando, A.; Carlton, E. 2010 Food Safety Survey: Key Findings and Topline Frequency Report. Center for Food

Safety and Applied Nutrition, U.S. Food and Drug Administration, Silver Spring, MD, USA, 2010. Available online: https://www.fda.gov/ (accessed on 17 October 2023).
13. Lando, A.; Verrill, L.; Wu, F.
FDA's Food Safety and Nutrition Survey (FSANS): 2019 Survey Report. Center for Food Safety and Applied Nutrition, U.S. Food and Drug Administration, Silver Spring, MD, USA, 2021. Available online: https://www.fda.gov/food/cfsan-consumer-behavior-research/food-safety-and-nutrition-survey-fsans (accessed on 17 October 2023).
14. European Commission.
Special Eurobarometer 505: Making Our Food Fit for the Future – Citizens' Expectations. Publications Office of the European Union, Luxembourg, 2020. ISBN 978-92-76-23035-9. https://doi.org/10.2875/826903
15. Cates, S.C.; Thomas, E.; Kosa, K.; Chapman, B.; Shelley, L.; Goulter, R.M.; Duong, M.; Kirchner, M.; Goodson, L.; Everhart, S.; Doring, L.; Jaykus, L.-A.
Food Safety Consumer Research Project: Meal Preparation Experiment Related to Thermometer Use. RTI International and North Carolina State University, Research Triangle Park, NC, USA, 2018. Available online: https://www.rti.org (accessed on 17 October 2023).
16. Cates, S.C.; Brophy, J.E.; Shumaker, E.; Hayes, M.; Chapman, B.
Food Safety Consumer Research Project: Web-Based Survey on Food Safety Behaviors and Consumer Education. RTI International and North Carolina State University, Research Triangle Park, NC, USA, 2020. Available online: https://www.rti.org (accessed on 17 October 2023).
17. safefood.
Food Safety Behaviour on the Island of Ireland. Volume 1: Food Safety Issues and Influences on Behaviour. safefood, Cork/Dublin, Ireland, 2011. Available online: https://www.safefood.net (accessed on 17 October 2023).
18. Food Standards Scotland.
Food in Scotland Consumer Tracking Survey Wave 9: Food Safety & Authenticity Module – Summary Report. Food Standards Scotland, Aberdeen, UK, 2020. Available online: https://www.foodstandards.gov.scot (accessed on 17 October 2023).
19. Redmond, E.C.; Curnin, A.; Day, C.; Morris, J.; Eves, A.; Raats, M.
Systematic Review of the Relative Proportion of Foodborne Disease Associated with Food Preparation or Handling Practices in the Home. Food Standards Agency, London, UK, 2018. Available online: https://www.food.gov.uk (accessed on 17 October 2023).
20. GfK NOP.
A Quantitative Assessment of Consumers' Attitudes towards Raw Meat Decontamination Treatments (FS241052). Food Standards Agency, London, UK, 2013. Available online: https://www.food.gov.uk (accessed on 17 October 2023).
21. Food Standards Agency.
COVID-19 Consumer Tracker Survey: Summary Report (Waves 1–19). Food Standards Agency, London, UK, 2022. https://doi.org/10.46756/sci.fsa.gnu416
22. Armstrong, B.; King, L.; Clifford, R.; Jitlal, M.; Mears, K.; Parnell, C.; Mensah, D.
Food and You 2: Wave 6 Key Findings. Food Standards Agency, London, UK, 2023. https://doi.org/10.46756/sci.fsa.djj797
23. European Commission; European Food Safety Authority (EFSA).
Special Eurobarometer 97.2: Food Safety in the EU. European Union, Luxembourg, 2022. ISBN 978-92-9499-467-7. https://doi.org/10.2805/729388

24. U.S. Food and Drug Administration (FDA); Center for Food Safety and Applied Nutrition. Food Safety and Nutrition Survey (FSANS): 2019 Survey Report. FDA, Silver Spring, MD, USA, 2021. Available online: https://www.fda.gov/food/cfsan-consumer-behavior-research/food-safety-and-nutrition-survey-fsans (accessed on 17 October 2023).
25. Centers for Disease Control and Prevention (CDC). Use of a Self-Assessment Questionnaire for Food Safety Education in the Home Kitchen—Los Angeles County, California, 2006–2008. *MMWR Morb. Mortal. Wkly. Rep.* 2010, *59*, 1098–1101. Available online: https://www.cdc.gov/mmwr/preview/mmwrhtml/mm5934a2.htm (accessed on 17 October 2023).
26. U.S. Food and Drug Administration (FDA). Food Safety Survey: Consumer Food Handling Practices. FDA, Silver Spring, MD, USA, 2016. Available online: https://www.fda.gov/media/101366/download (accessed on 17 October 2023).
27. Food Standards Australia New Zealand (FSANZ). Consumer Attitudes Survey. FSANZ, Canberra, Australia, 2010. Available online: https://www.foodstandards.gov.au/publications/documents/Consumer%20Attitudes%20Survey.pdf (accessed on 17 October 2023).
